# Screening of Novel Antimicrobial Diastereomers of Azithromycin–Thiosemicarbazone Conjugates: A Combined LC-SPE/Cryo NMR, MS/MS and Molecular Modeling Approach

**DOI:** 10.3390/antibiotics11121738

**Published:** 2022-12-02

**Authors:** Iva Habinovec, Ivana Mikulandra, Paula Pranjić, Saša Kazazić, Hana Čipčić Paljetak, Antun Barišić, Branimir Bertoša, Mirjana Bukvić, Predrag Novak

**Affiliations:** 1 Department of Chemistry, Faculty of Natural Sciences, University of Zagreb, Horvatovac 102a, HR-10000 Zagreb, Croatia; 2 Ruđer Bošković Institute, Bijenička Cesta 54, HR-10000 Zagreb, Croatia; 3 Center for Translational and Clinical Research, School of Medicine, University of Zagreb, Šalata 3, HR-10000 Zagreb, Croatia; 4 Selvita, Prilaz baruna Filipovića 29, HR-10000 Zagreb, Croatia

**Keywords:** azithromycin conjugates, macrozones, diastereomers, antibacterial activity, HPLC-SPE/cryo NMR, MS/MS, molecular modeling

## Abstract

A well-known class of antibacterials, 14- and 15-membered macrolides are widely prescribed to treat upper and lower respiratory tract infections. Azithromycin is a 15-membered macrolide antibiotic possessing a broad spectrum of antibacterial potency and favorable pharmacokinetics. Bacterial resistance to marketed antibiotics is growing rapidly and represents one of the major global hazards to human health. Today, there is a high need for discovery of new anti-infective agents to combat resistance. Recently discovered conjugates of azithromycin and thiosemicarbazones, the macrozones, represent one such class that exhibits promising activities against resistant pathogens. In this paper, we employed an approach which combined LC-SPE/cryo NMR, MS/MS and molecular modeling for rapid separation, identification and characterization of bioactive macrozones and their diastereomers. Multitrapping of the chromatographic peaks on SPE cartridges enabled sufficient sample quantities for structure elucidation and biological testing. Furthermore, two-dimensional NOESY NMR data and molecular dynamics simulations revealed stereogenic centers with inversion of chirality. Differences in biological activities among diastereomers were detected. These results should be considered in the process of designing new macrolide compounds with bioactivity. We have shown that this methodology can be used for a fast screening and identification of the macrolide reaction components, including stereoisomers, which can serve as a source of new antibacterials.

## 1. Introduction

A well-known class of antibacterials, 14- and 15-membered macrolides are widely prescribed to treat upper and lower respiratory tract infections [1]. Azithromycin is a semisynthetic 15-membered macrolide antibiotic derived from erythromycin, possessing a broad spectrum of antibacterial potency and favorable pharmacokinetics [2]. It exerts its activity by binding to the bacterial ribosomal 23S rRNA in domain V at the peptidyl transferase region and blocks the exit tunnel, which in turn inhibits bacterial protein synthesis [3,4,5]. Bacterial resistance to marketed antibiotics is growing rapidly and represents one of the major hazards to human health worldwide. There are several mechanisms of bacterial resistance to macrolides, but modification of the ribosomal binding sites and efflux pumps are the most common [6]. Today, there is a high need for discovery of new anti-infective agents to combat resistance. Novel conjugates of azithromycin and thiosemicarbazones, the macrozones, represent one such type of compounds that exhibit promising activities against resistant bacterial strains [7,8]. Recently, we reported on design, synthesis and biological evaluation of several macrozone classes displaying activities against bacteria to which azithromycin was inactive [7]. In this work, we have employed a combination of the LC-SPE/cryo NMR and MS/MS methodologies [9,10,11] to screen, identify and characterize main reaction outcome components of the novel macrozones, which also included diastereomers as a potential new source of bioactive compounds. These hyphenated systems have proven very useful in screening complex mixtures of compounds of biological [12] and pharmaceutical [13,14,15] relevance since they could provide important and reliable information on the identity and structure of each component in a short time.

**Scheme 1:**
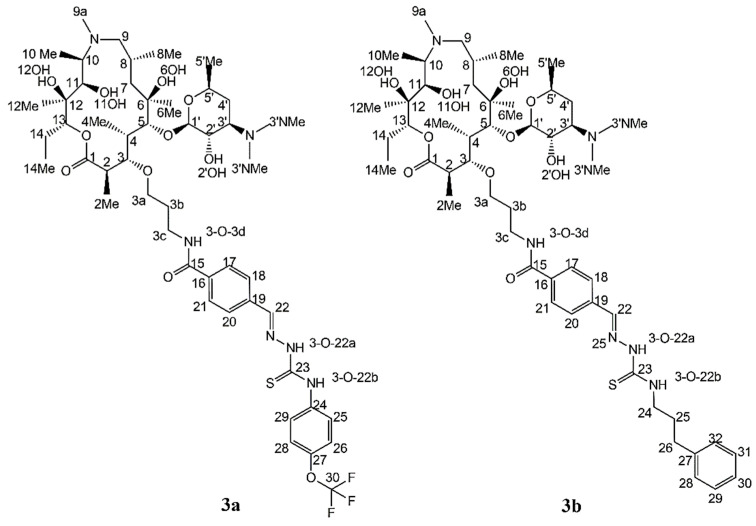
The structure of 3-macrozones **3a** and **3b**.

Recent technological developments such as cryo-platforms, capillary NMR with microcoil probes and the introduction of solid phase extraction (SPE) into an LC-SPE-NMR system have made it possible to detect compounds present at a very low concentration level [16,17,18]. Fully hyphenated LC-SPE-NMR or hyphenated LC-SPE in combination with cryo NMR, e.g., LC-SPE/cryo NMR, today represent widely accepted tools for dereplication and discovery of complex natural product mixtures, metabolites and bioactive and drug substances. In the present work, we prepared novel macrozones (Scheme 1) and applied LC-SPE/cryo NMR in combination with MS/MS and molecular modeling for their rapid identification and structural characterization and screened them against a panel of Gram-positive and Gram-negative bacteria, including macrolide-resistant strains.

Surprisingly, when analyzing MS/MS data of the reaction mixture components, we observed several peaks with precursor ions of the same *m*/*z* ratio for both macrozones, which pointed toward the presence of isomers. Hence, we were keen to explore what the nature and structures of those isomers were by using NMR and molecular modeling and whether they would exhibit similar or different bioactivity as the main compounds.

## 2. Results and Discussion

**Figure 1:**
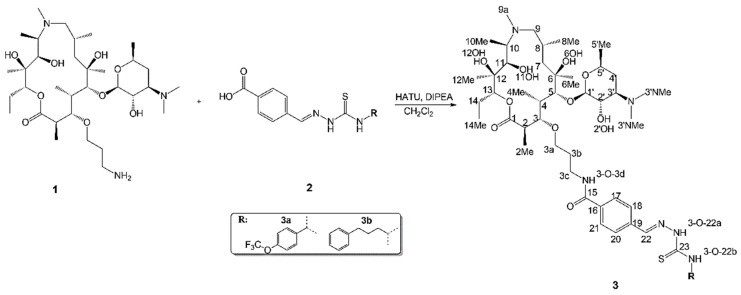
The final step in the synthesis of 3-macrozones.

The final synthetic step for obtaining macrozones **3a** and **3b** is displayed in Figure 1. The synthetic routes for obtaining aminopropyl azithromycin and thiosemicarbazones have been reported earlier [7,8].

**Figure 2:**
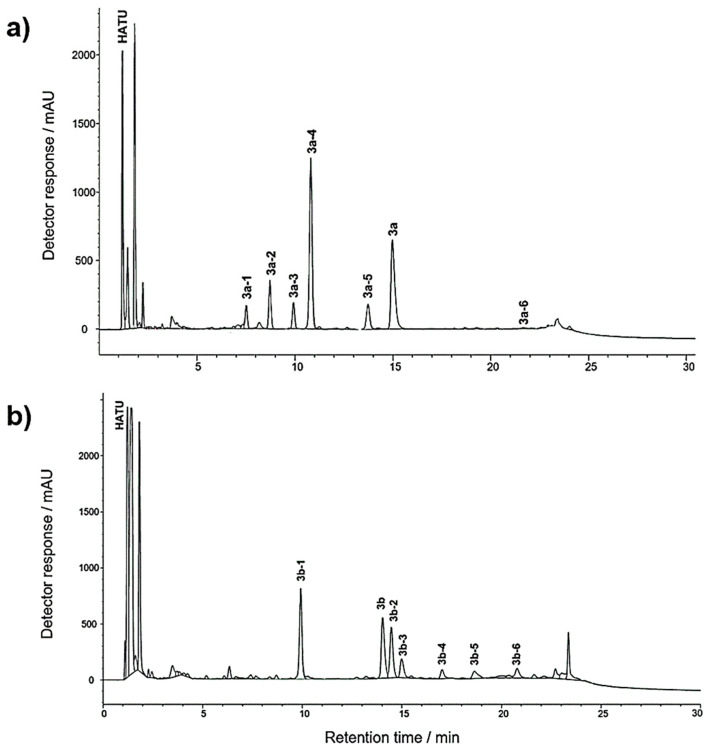
LC chromatogram of reaction mixture components of (**a**) **3a**-macrozone and (**b**) **3b**-macrozone.

Prior to structural characterization by NMR and MS, chromatographic separation of the main reaction components was optimized, and the LC chromatograms are shown in Figure 2. MS analysis has readily pointed toward several components with the same molecular ion peaks for both **3a** and **3b,** reflecting the existence of isomeric forms. Hence, it was of importance to reveal how isomerization occurred and whether the isomers would display different biological activity acting as a source of novel bioactive compounds. In the chromatogram, some other unknown components are detected as well (Figure 2). We employed here a hyphenated LC-DAD-SPE methodology to separate and concentrate the components of interest for NMR structure determination and test them against a panel of bacterial strains.

### 2.1. SPE Preconcentration

After chromatographic peaks were separated on the LC column, they were sent to SPE to be concentrated. Extraction efficiency was tested by using several SPE-cartridges with different stationary phases (see Section 3) and most of the reaction mixture components were well-retained at C18 SPE-cartridges. In order to obtain sufficient quantities for NMR analysis and biological evaluation, a multiple trapping option was used [18] at a make-up flow rate of 1.5 mL min^−1^ for most components and 3 mL min^−1^ for more polar ones. Subsequently, samples were eluted into 3 mm NMR tubes, and cryo NMR spectroscopy was used to record one- and two-dimensional spectra for structure determination.

### 2.2. Structure of Identified Reaction Components Not Related to Isomers

**Figure 3:**
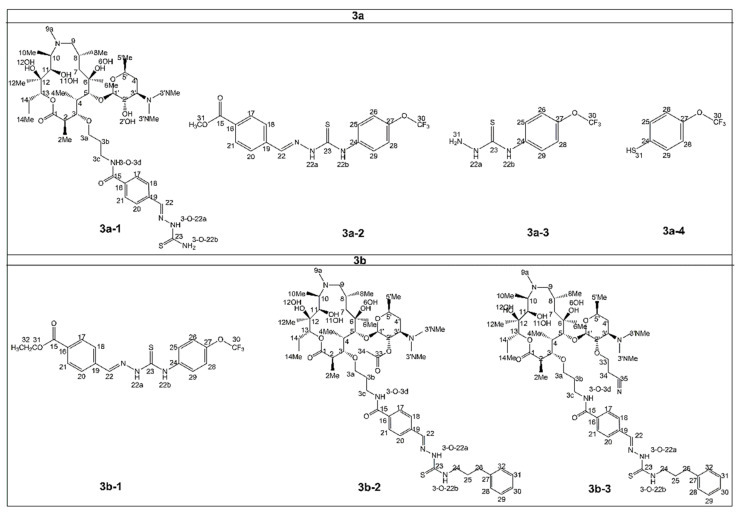
Structures of reaction components not related to stereoisomers.

A close inspection of one- and two-dimensional NMR spectra of the component **3a-1** suggested a lack of a trifluoromethoxy benzyl moiety in the structure. All other spin–spin connectivities were found in HSQC and HMBC spectra (Supplementary Materials Figures S1–S3). MS spectrum (Figure S4) displayed peaks that were in accordance with the proposed structure of **3a-1** shown in Figure 3. Proton spectra of **3a-2**, **3a-3** and **3a-4** (Figure S5) indicate that these compounds have thiosemicarbazone-related structures as depicted in Figure 3. A precursor ion for **3a-2** was detected at *m*/*z* 398 (Figures S6–S11). Similarly, a comparison of NMR and MS data for compound **3b-1** (Figures S12–S15) revealed the thiosemicarbazone structural fragment. NMR and MS spectra of compounds **3b-2** and **3b-3** reflect structural similarities with the parent compound **3b**. Several additional peaks were observed in proton, HSQC and HMBC spectra of **3b-2** (Figures S16–S18), revealing an acetyl group at position 2′ (Figure 3). The observed molecular ion peak in the MS spectrum at *m*/*z* 1013.6 is in accordance with the mass of acetylated macrozone (Figure S19). A close inspection of proton, HSQC and HMBC spectra of **3b-3** (Figures S20–S24) pointed toward an additional ethylene -CH_2_-CH_2_-R moiety attached to the position C2′ (Figure 3). MS spectrum detected a precursor ion peak at *m*/*z* 1024.6 and product ion at *m*/*z* 971.6, which corresponds to a loss of a propionitrile group (Figure S25).

### 2.3. Structure of Stereoisomers

**Figure 4:**
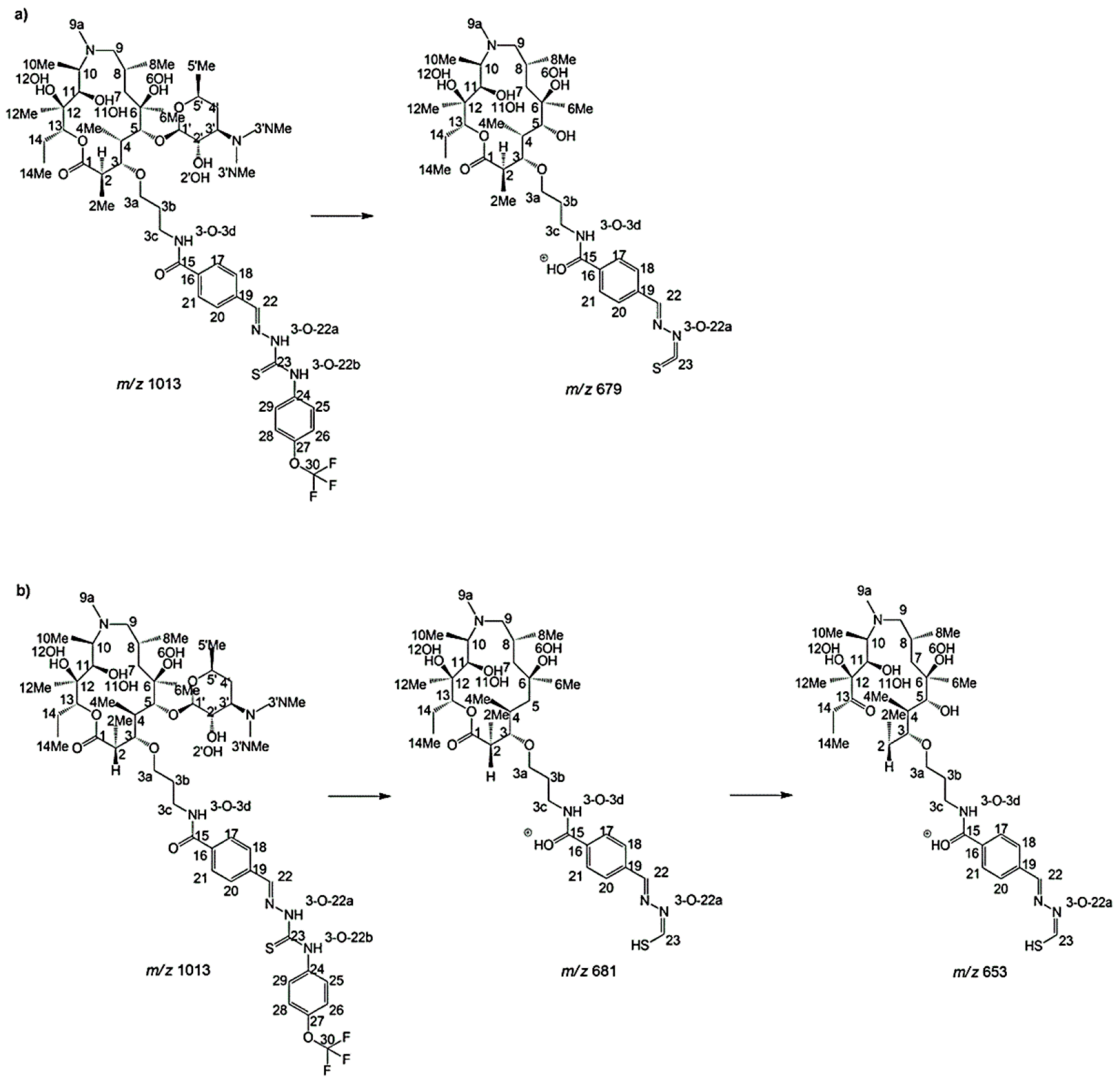
Fragmentation pattern observed in the MS/MS spectra of macrozones (**a**) **3a** and (**b**) **3a-5**.

After preconcentration on SPE cartridges, compounds **3a-5** and **3a-6** (Figure 2a) were sent to NMR and MS for structure determination. MS spectra of both compounds displayed precursor ion peaks at *m*/*z* 1013, which was also found for the main compound **3a**, corresponding to the same molecular masses. Subsequently, MS/MS experiments exhibited a similar fragmentation pattern for all three compounds, pointing toward diastereomeric structures (Figure 4).

**Figure 5:**
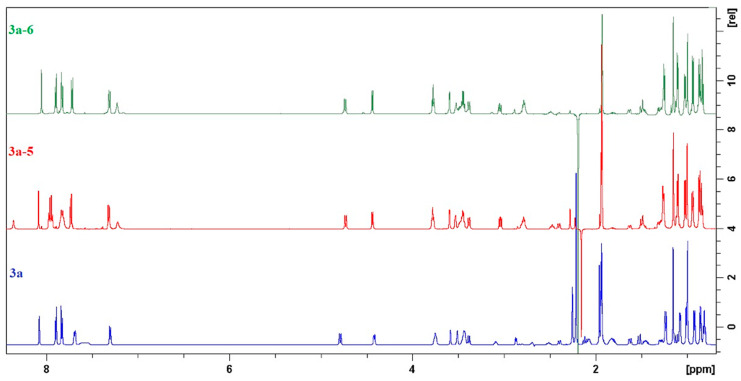
Proton NMR spectra of **3a** (blue), **3a-5** (red) and **3a-6** (green).

In the initial fragmentation step, the product ion at *m*/*z* 836.5 appeared first, which was further fragmented to ions at *m*/*z* 679 and 681 as a consequence of a loss of a desosamine sugar unit. These two ions differ in intensities for isomers, pointing toward inversion of carbon atom in this sugar unit. Similar changes in fragment-peak intensities were observed in the MS spectrum of **3a-6** (Figure S26). Proton NMR spectra of **3a**, **3a-5** and **3a-6**, displayed in Figure 5, and two-dimensional COSY, HSQC and HMBC spectra (Figures S27–S35), are in accordance with those observations, reflecting high structural similarities and confirming the existence of diastereomers.

We have recently reported that the precursor for obtaining 3-macrozones, e.g., 3-aminopropyl azithromycin, adopted a classical *folded-out* conformation characteristic of related 15-membered macrolides [8]. As previously demonstrated in the literature, macrolides usually existed in the two main conformations in solution, e.g., *folded-in* and *folded-out*, referring to the inward or outward folding of the ring fragment C2–C5. Large vicinal coupling constants ^3^*J*_H2_,_H3_ (≈10 Hz) and NOE proton–proton contacts H4-H11 indicate *folded-out* conformation, while much lower ^3^*J*_H2,H3_ values (≈2–3 Hz) and a close contact of atoms H3 and H11 are characteristic of the *folded-in* conformer [8,19]. The ^3^*J*_H2_,_H3_ values of 10.12 Hz and 10.05 Hz were obtained for **3a** and **3b**, respectively. Furthermore, for both macrozones, the H4–H11 NOE cross peak was found in the NOESY spectra, while H3–H11 was missing. These observations are in accordance with the *folded-out* conformation. The isomers **3a-5**, **3a-6** and **3b-6** all displayed ^3^*J*_H2_,_H3_ values of about 10 Hz (Table S1), being in accordance with *folded-out* conformation, which means that inversion of chirality in those isomers did not influence folding of the macrocyclic ring.

**Figure 6:**
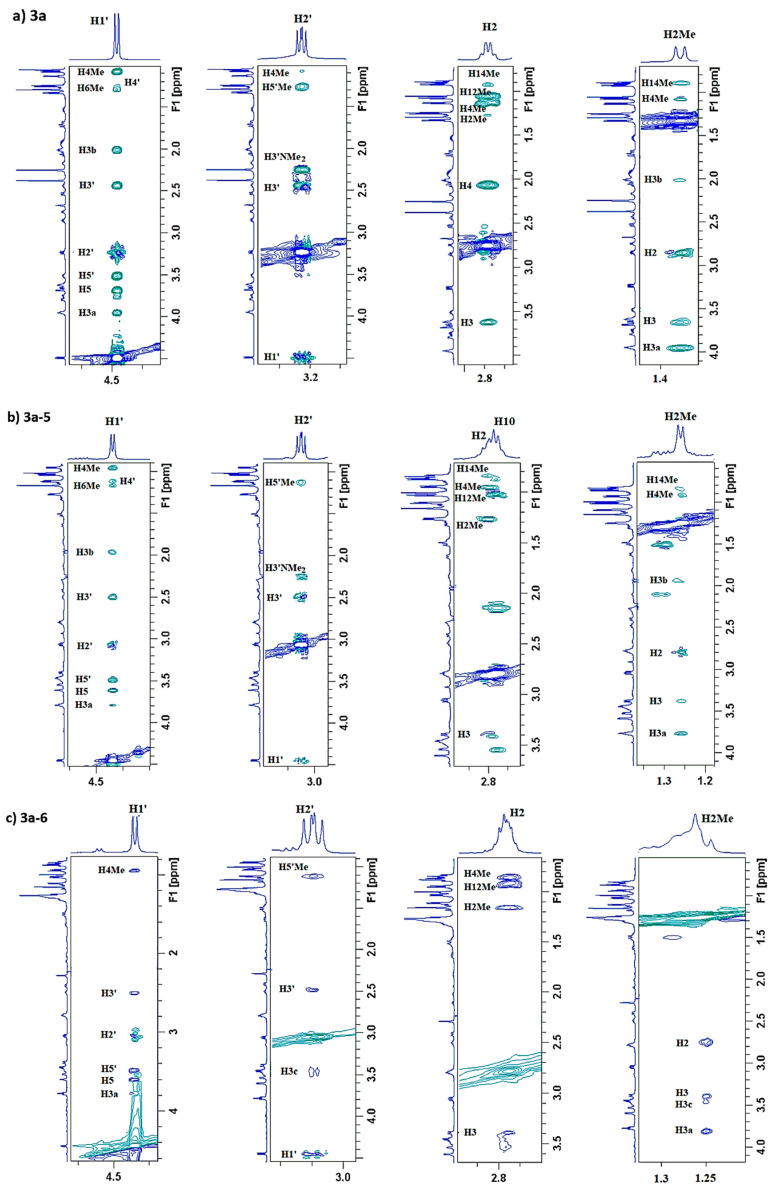
Narrow regions of the NOESY spectra of (**a**) **3a**, (**b**) **3a-5** and (**c**) **3a-6** for atoms H-1′, H-2′, H-2 and H-2Me.

In order to decipher the carbon site of inversion, we further used NOESY NMR experiments coupled with molecular dynamic (MD) simulations. The overlaid NOESY spectra of **3a, 3a-5** and **3a-6** (Figure 6) shows some indicative changes in the NOE cross peaks of the protons H-2, H-2Me, H-1′and H-2′. All other proton–proton NOE contacts were similar.

The H2-H14Me cross peak was detected in the spectra of **3a** and **3a-5** but was missing in the spectrum of **3a-6**. Furthermore, it is clearly seen in Figure 6 that H-2Me exhibited a close proximity to atoms H-14 and H-4Me in **3a** and **3a-5** but not in **3a-6**, where no corresponding NOE cross peaks were found. NOE contacts of proton H-1′ were the same for isomers **3a** and **3a-5**, while the **3a-6** cross peaks of H-1′ with H-6Me and H-3b were not present, as was the case in the other two isomers (Figure 6).

**Figure 7:**
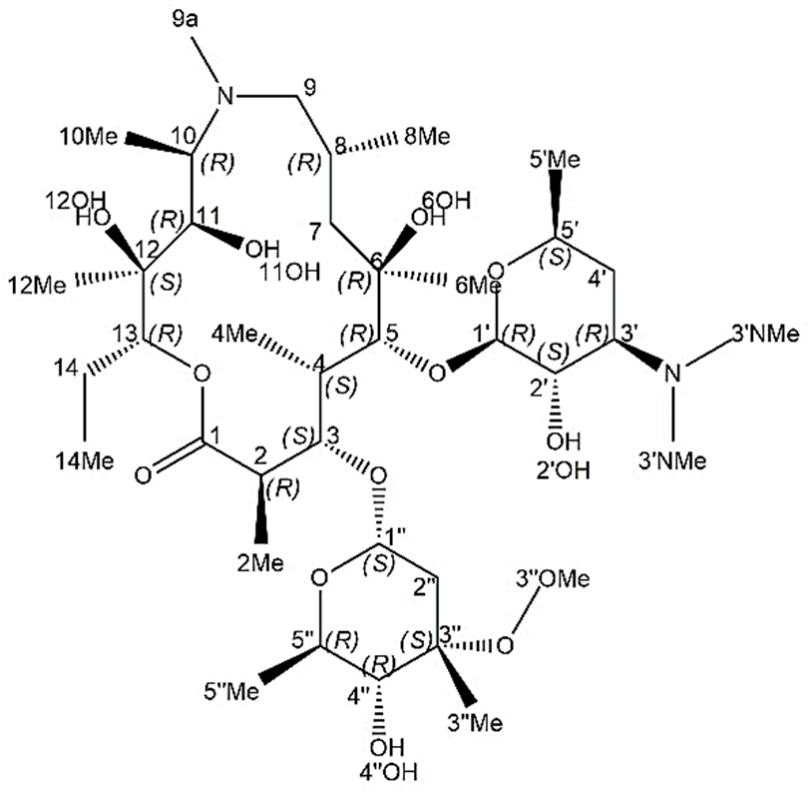
Absolute configuration for azithromycin.

Originally, the configurations at C-2, C-2′ and C-1′ in azithromycin are R, S and R, respectively (Figure 7), and we expect that the main compound **3a** has the same configuration.

**Figure 8:**
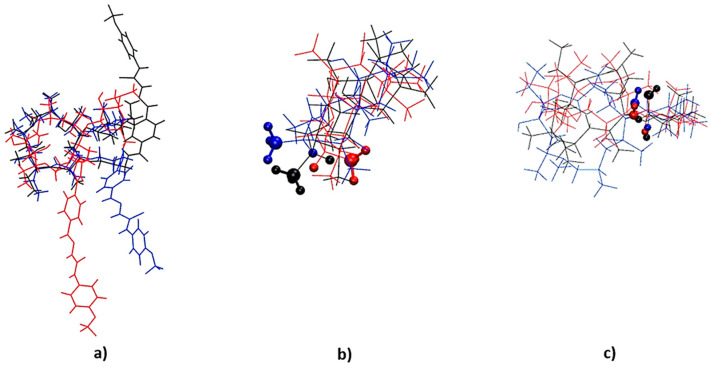
(**a**) The overlapped conformations of **3a** (black), **3a-5** (red) and **3a-6** (blue), (**b**) highlighted configurations at C-2 and (**c**) highlighted configurations at C-1′.

Consequently, changes in NOESY connectivities observed for H-2, H-2Me, H-2′ and H-1′ serve as a sound argument for an assumption that the inversion took place at positions 2, 1′ and 2′. In order to provide further evidence to support these observations, we performed molecular dynamics (MD) simulations for the compounds and diastereomers of interest. The goal of MD simulations was to provide insight into the intramolecular distances that could help resolve the doubts about the presence of different stereoisomers in solution. The analyses of MD simulations showed that for an R configuration at C-2, the average distance between atoms H-2 and H14Me is around 4.2 nm, while for an S configuration, it is around 5.2 nm (Table S2 and Figures S36–S39). Hence, one can expect the NOE cross peak H2-H14Me for an R arrangement, which was actually observed in the NOESY spectra of **3a** and **3a-5** but not of **3a-6**, suggesting an S configuration for this isomer. NOE correlations of H-2Me support this conclusion. Namely, cross peaks of H-2Me with H-14Me and H-4Me were observed for **3a** and **3a-5** but not for **3a-6** (Figure 6). Superimposed conformations sampled during the MD simulations of **3a**, **3a-5** and **3a-6** are depicted in Figure 8.

MD calculations indicate contact of H-2′ and H-4Me for an S configuration at C-2′ (around 4.4 nm, Table S3 and Figures S40 and S41), which should lead to a weak NOE cross peak in the spectrum. This peak of low intensity was actually observed for **3a** but not for **3a-5** and **3a-6**, pointing toward S configuration in **3a** and R configuration in **3a-5** and **3a-6** at that carbon atom. Additionally, contact between protons H-2′ and H-3c was observed in **3a-6,** while in the corresponding spectra of **3a** and **3a-5,** that contact was not found. According to simulations, a characteristic of an R arrangement at C-1′ should be NOE contact between H-1′ and methyl protons H-6Me, owing to their close proximity (around 3.6 nm, Figure S42). Indeed, this correlation was observed in the NOESY spectra of **3a** and **3a-5** and was missing in the spectrum of **3a-6**. The same was true for NOE cross peaks between H-1′ and H-3b, which also was not found for **3a-6**. This suggests that isomers **3a** and **3a-5** most probably adopt R configuration at C-1′, while S configuration prevails for **3a-6** (Table 1)

As previously mentioned, in the synthesis of macrozone **3b**, compounds **3b-2** and **3b-3** are 2′-acetyl and 2′-propionitrile derivatives of the main compound (Figure 3) and also show antibacterial activity (Table 2). MS spectra of **3b-4**, **3b-5** and **3b-6** indicate stereoisomeric structures. Unfortunately, compounds **3b-4** and **3b-5** could not be trapped in sufficient concentration to allow for a reliable NMR analysis. The concentration of isomer **3b-6** was also not very good, but we managed to record and analyze the NOESY spectrum. In analogy to **3a** isomers, characteristic NOE peaks of H-2 and H-2Me resembled those found for **3a** and **3a-5,** as well as for **3b,** as an indication of R configuration at C-2. Subsequently, NOE cross peaks involving atoms H-1′ and H-2′ were found to be similar to those observed for **3a-6,** reflecting S and R arrangements, respectively (Figures S43 and S44). Hence, we proposed the following relative configurations of **3a** and **3b** diastereomers (Table 1.)

We further intend to elaborate and explain possible reasons for the formation of diastereomers. Why would inversion of chirality occur at positions C-2, C-1′ and C-2′? The extreme sensitivity of the macrolides to both acidic and basic conditions presents a challenge with regard to effecting both chemical and stereochemical modifications. The 3-macrozones were achieved from azithromycin by a sequence of seven steps [7]. Potential lability to epimerization of the β-keto ester moiety was already observed in macrolide-class ketolides [20]. The C2-position in the ketolides is chemically activated by the adjacent oxo groups and could be readily enolized. The ketolides, as enolates, react readily with electrophilic reagents (halogenation, fluorination, etc.). Although **3a** macrozone exists primarily in the natural C-2 *R* configuration, its S epimer (**3a-6**) has also been detected in the reaction mixture but in much smaller amounts. Furthermore, during the preparation of 3-aminopropyl-azithromycin, the precursor for obtaining macrozones [7], several chemical transformations could affect conversion of stereochemistry at some positions. So, it is likely that the inversions took place at the reaction steps when application of strong bases is necessary, such as O-alkylation at position C-3 and protection/deprotection of the 2′-OH group (Figure 1). Hence, most probably, epimerization processes at C-2 and C-2′ are a result of a base-mediated racemization involving a deprotonation/protonation step, through an enolate intermediate. The acidic nature of the lactone α-carbonyl hydrogen at C-2 and use of NaH as a base during chemical synthesis are responsible for the appearance of stereoisomers. Most likely, in the same reaction conditions, inversion of stereochemistry occurred at position C-1′.

### 2.4. Antibacterial Evaluation

The main compounds **3a** and **3b** together with diastereomers and derivatives **3b-2** and **3b-3** were screened in vitro against a panel of Gram-positive and Gram-negative bacteria using the standard microdilution method for determination of minimal inhibitory concentrations (MICs). MIC values of these compounds along with the standard antibiotic azithromycin are displayed in Table 2.

As seen from the Table 2, macrozone **3a** exhibits somewhat better overall antibacterial activity compared to **3b**. Compared to azithromycin, both compounds showed improved activity against constitutively resistant strains, as well as similar or better potency against efflux-resistant Gram-positive strains (*S. pneumoniae*, *S. pyogenes* and *S. aureus*). It is interesting to note that variation in biological activity was also observed among epimers. Thus, compared to **3a**, the epimer **3a-5** was less active against efflux-resistant *S. pneumoniae* and *S. pyogenes* but more active against constitutive-resistant *S. aureus.* The same was true for **3a-6,** which additionally showed better activity against efflux-resistant *S. aureus* than **3a** and **3a-5** (Table 2). The isomer **3b-6** exhibited lower activities compared to the main compound **3b**. These results demonstrate that slight changes in conformations among epimeric structures influence biological activity, and this might be important for further derivatization of macrolide scaffolds in the search for better ribosome inhibitors.

## 3. Materials and Methods

### 3.1. Materials and Reagents

**3a**-macrozone and **3b**-macrozone were synthesized in our laboratory according to the procedure described in a previously published article [7]. Acetonitrile (HPLC-grade) was purchased from Fisher Scientific (Loughborough, UK). Ammonium bicarbonate (HPLC-grade) was obtained from Sigma-Aldrich (St. Louis, MO, USA). Deionized water was produced by Millipore Milli-Q Advantage A10 purification system (Molsheim, France). Deuterated acetonitrile (99.8%-D) was procured from Euriso-Top SAS (Saint-Aubin Cedex, France). Acetonitrile (MS-grade) was purchased from Carlo Erba (Val de Reuil, France).

### 3.2. Sample Preparation

Reaction mixtures of **3a**- and **3b**-macrozone were evaporated to dryness and stored at 4 °C. The synthetic routes and procedures for the preparing of aminopropyl azithromycin and thiosemicarbazones are given in Supplementary (Figures S45–S47). Before LC-SPE analysis, samples were dissolved in acetonitrile at a concentration of 18.2 mg mL^−1^ for **3a**-macrozone and 23.3 mg mL^−1^ for **3b**-macrozone. For the MS analysis, dry extracts of isolated components were prepared by dissolving in MS-grade acetonitrile. Mass concentration ranged from 0.13 µg mL^−1^ to 0.28 µg mL^−1^ for **3a** and from 0.05 µg mL^−1^ to 0.43 µg mL^−1^ for **3b**, respectively.

### 3.3. Liquid Chromatography

For the chromatographic analysis, an Agilent 1260 Infinity HPLC system was used. The HPLC system consisted of quaternary pump G1311B, an autosampler G1329B, thermostated column compartment G1316A and a photo diode array detector G1315D. The chromatographic data were collected and analyzed by OpenLab CDS Chemstation A.01.08.108. Chromatographic separation of the **3a**- and **3b**-macrozone reaction mixture components was optimized as described in a previously published paper [16].

Components were efficiently separated on a Waters XBridge Phenyl column using a gradient elution with acetonitrile (A) and ammonium bicarbonate solution (10 mM) corrected to pH 10 with ammonia solution (B). The gradient elution program was as follows: 0–20 min (50% A–68% A), 20–21 min (68% A–100% A), 21–30 min (100% A), 30–30.1 min (100% A–50% A) and 30.1–35 min (50% A). Flow rate was 1.000 mL min^−1^. Column temperature was 25 °C. Injection volume was 15 µL for **3a** and 8 µL for **3b**, while detection wavelength was 210 nm.

### 3.4. On-Line HPLC-SPE

For the postcolumn solid-phase extraction of the macrozone reaction mixture components, a Prospekt 2 SPE unit (Spark Holland, Emmen, The Netherland) was used. Analytes were trapped on HySphere C18 HD SPE-cartridges (2 mm × 10 mm). Trapping of the analytes was performed in the multitrapping mode. Threshold absorbance levels were set at 210 nm. Sample injection volumes were 15 µL and 8 µL. Solution B was used as a make-up solvent with a flow rate of 1.5 mL min^−1^. After the peak trapping, SPE-cartridges were dried in nitrogen stream for 59 min. Components were eluted from SPE-cartridges to 3 mm NMR tubes with 150 µL of acetonitrile-d3 and NMR spectra were recorded. For collection and analysis of the LC-SPE data, HyStar 3.2 software was used.

### 3.5. NMR Spectroscopy

NMR spectra with an NOESY-type solvent suppression module were recorded on Bruker Avance NEO 600 MHz spectrometer equipped with inverse TCI Prodigy cryoprobe and z-gradient accessories. After LC-SPE analysis, isolated components were eluted from SPE-cartridges with acetonitrile-d3 into 3 mm NMR tubes. In order to adapt 3 mm tubes to 5 mm Bruker spinners, MATCH inserts were used. All experiments were carried out at 298 K. TMS was used as an internal standard.

Proton spectra parameters were as follows: 128–256 scans, depending on the concentration of analyte, spectral width 11,904 Hz and digital resolution 1.45 Hz per point. In the gCOSY experiment, 2048 points in the *f*2 dimension and 256 increments in the *f*1 dimension were used. For each increment, 16–32 scans and the spectral width of 9615 Hz were applied. Digital resolution was 9.39 Hz and 75.12 Hz per point in the *f*2 and *f*1 dimensions, respectively. NOESY spectra parameters were as follows: 2048 points in the *f*2 dimension, 512 increments in the *f*1 dimension, 72–144 scans for each increment, spectral width of 9615 Hz, relaxation time delay 2 s and mixing time 400 ms.

The gHSQC spectra were acquired with 32–100 scans. For gHSQC experiment parameters were as follows: spectral width 9615 Hz in the *f*2 and 27,166 Hz in the *f*1 dimension. For gHSQC and gHMBC spectra, 4 K data points were applied in the time domain and 256 increments were collected for each data set. The gHMBC spectra were acquired with 40–160 scans using the spectral width of 9090 Hz in the *f*2 domain and 33,204 Hz in the *f*1 domain.

### 3.6. MS Analysis

MS analysis was performed on an amaZon ETD ion trap mass spectrometer (Bruker Daltonik, Bremen, Germany) equipped with the standard ESI ion source. MS parameters were as follows: nebulizer pressure was 8 psi, drying gas flow rate was 5 L min^−1^ and drying gas temperature was 200 °C. Nitrogen was used as a drying and nebulizing gas. For the MS analysis, samples were dissolved in 1 mL of MS-grade acetonitrile at a concentration ranging from 0.05 mg mL^−1^ to 0.43 mg mL^−1^. Before the MS analysis, samples were diluted 1000 times in electrospray solution (acetonitrile/water with 0.1% formic acid, 50/50, *v*:*v*) and injected into the ESI source by a syringe pump at a flow rate of 1 μL min^−1^. MS spectra were recorded in positive polarity mode with the potential on the capillary cap of 4500 V. Helium was used as the collision gas. Full-scan ESI-MS spectra were obtained in a positive ion acquisition mode ranging from 100 *m*/*z* to 1500 *m*/*z*. For the tandem MS spectra, collision-induced dissociation (CID) fragmentation was performed. Parent ions were isolated in a +/− 2 Da wide *m*/*z* window. Collision energy for the fragmentation of the parent ions was 0.4 V.

### 3.7. Molecular Dynamics Simulations

Compounds: **3a**, **3a-5**, **3a-6**, **3b** and **3b-6** were generated in silico using the Maestro program package and prepared for molecular dynamics (MD) using the GROMACS 2020.4 program package [21]. Compounds were parameterized using LigParGen server + and OPLS-AA forcefield. Each compound was solvated in acetonitrile using an explicit solvent model. Forcefield parameters for acetonitrile were obtained using the OPLS-AA library. All calculations were conducted using the GROMACS 2020.4 program package. Van der Waals interactions were calculated using Lennard-Jones potential. Long-range electrostatic interactions were calculated using Particle-mesh Ewald (PME) with real space Coulomb interactions cut off at 1.2 nm using a Fourier spacing of 0.12 nm and Verlet cut-off scheme. Periodic boundary conditions (PBC) were applied. Prior to MD simulations, each system was energy minimized using the steepest descent algorithm until convergence criterium was achieved (E = 1000 kJ mol^–1^ nm^–1^). The equilibration phase of MD simulation was conducted using a leap-frog algorithm for integrating Newton’s equations of motion. Equilibration was divided in two phases, NTV and NPT ensemble. During the first 600 ps of NVT equilibration, the temperature was gradually increased from 10 K to 300 K using a Berendsen thermostat [22]. During the next 500 ps of NPT equilibration, temperature (298.15 K) and pressure (1 bar) were kept constant using a Berendsen thermostat and barostat [23]. The production phase of each MD simulation was conducted in the NPT ensemble using a velocity Verlet algorithm for integrating Newton’s equations of motion. Production phase was 500 ns with a time step 1 fs. Constant temperature (298.15 K) was maintained via Nosé-Hoover thermostat [24] with a coupling constant of 1.0 ps^−1^. Pressure was set to 1 bar and was controlled with a Martyna–Tuckerman–Tobias–Klein (MTTK) barostat [25] with a time constant for pressure coupling of 5 ps^−1^. Analyses of trajectories were performed using Gromacs analyzing tools and the VMD program [26].

### 3.8. Antibacterial Activity

Minimum inhibitory concentrations (MICs) were determined by the broth microdilution method according to guidelines of the Clinical Laboratory Standards Institute (CLSI) in cation-adjusted Mueller–Hinton broth (MHB) or MHB supplemented with 5% horse serum for streptococci. Compounds were prepared as 10 mg mL^−1^ solutions in dimethyl sulfoxide (DMSO). Final dilutions of the tested compounds were prepared in testing medium in the 64–0.125 μg mL^−1^ concentration range with the highest DMSO concentration of 0.64%. Compounds were tested in duplicate. *E. coli* and *S. aureus* were grown on Mueller–Hinton agar plates (by Becton Dickinson GMBH, Hidelberg, Germany), *E. faecalis* and *M. catarrhalis* were grown on Mueller–Hinton agar plates supplemented with 5% sheep blood (BAP) in ambient air, while *S. pneumoniae* and *S. pyogenes* were grown on BAP in an atmosphere with 5% CO_2_. Inocula were prepared by the direct colony suspension method and microtiter plates were inoculated with 5 × 10^4^ CFU/well. Results were determined by visual inspection of microtiter plates after 20–22 h incubation at 37 °C in ambient air. The validity of the screen was confirmed by determining MIC values for the reference antibiotic, azithromycin.

## 4. Conclusions

We have demonstrated that by a combination of LC-SPE/cryo NMR, MS/MS and molecular modeling, a rapid screening of biologically active macrozone isomers can readily be achieved. This methodology has proven sensitive and useful for the isolation, identification and structural determination of bioactive reaction mixture components. Biological evaluation of different stereoisomers has demonstrated good antibacterial activities against resistant pathogens. The results obtained in this study can further be exploited in the process of design and discovery of macrolide compounds with activity against resistant bacteria.

## Figures and Tables

**Table 1:** Relative configurations at atoms C-2, C-1′ and C-2′ of bioactive macrozone stereoisomers.

Atom			
Isomer	C-2	C-1′	C-2′
**3a**	R	R	S
**3a-5**	R	R	R
**3a-6**	S	S	R
**3b**	R	R	S
**3b-6**	R	S	R

**Table 2:** In vitro antibacterial activity (MIC, μg/mL) for macrozones, macrozone derivatives and azithromycin. A diverse panel of clinical isolates with known mechanisms of macrolide resistance and standard strains were used.

Compound	*S. pneumoniae B0652*	*S. pneumoniae B0326*	*S. pneumoniae B0633*	*S. pyogenes B0542*	*S. pyogenes B0545*	*S. pyogenes B0544*	*S. aureus ATCC 29213*	*S. aureus B0331*	*S. aureus B0330*	*E. faecalis ATCC 29212*	*M. catarrhalis ATCC 23246*	*E. coli ATCC 25922*
	* eryS	M	cMLS	eryS	M	cMLS	eryS	M	cMLS			
	MIC (μg/mL)											
**3a**	8	8	64	2	8	32	8	16	32	8	4	>64
**3a-5**	16	32	64	16	16	32	16	16	8	16	2	>64
**3a-6**	4	16	64	4	16	32	16	8	16	64	8	>64
**3b**	2	16	64	4	16	64	8	8	16	32	4	>64
**3b-2**	2	16	64	4	16	64	8	16	16	32	4	>64
**3b-3**	16	64	>64	16	32	64	64	16	16	64	4	>64
**3b-6**	16	>64	>64	32	64	64	>32	16	16	>64	16	>64
Azithromycin	≤0.125	16	>64	≤0.125	16	>64	1	>64	>64	8	≤0.125	>64

* eryS—erithromycin sensitive; M—efflux resistance; cMLS—constitutive resistance to macrolide, lincosamine and streptogramin b antibiotics; ATCC—American Type Culture Collection.

## Data Availability

The data presented in this study are available within the article and in the Supplementary Materials.

## Supplementary Materials

The following supporting information can be downloaded at: https://www.mdpi.com/article/10.3390/antibiotics11121738/s1, Figure S1. Comparison of ^1^H NMR spectra of the macrozone 3a and component 3a-1; Figure S2. ^1^H-^13^C HSQC spectrum of the component 3a-1; Figure S3. ^1^H-^13^C HMBC spectrum of the component 3a-1; Figure S4. MS spectrum of the component 3a-1; Figure S5. Comparison of ^1^H NMR spectra of the reactant 2 (blue), component 3a-2 (red), component 3a-3 (green) and component 3a-4 (purple); Figure S6. MS spectrum of the component 3a-2; Figure S7. COSY spectrum of the component 3a-2; Figure S8. ^1^H-^13^C HSQC spectrum of the component 3a-2; Figure S9. ^1^H-^13^C HMBC spectrum of the component 3a-2; Figure S10. ^1^H-^13^C HMBC spectrum of the component 3a-3; Figure S11. ^1^H-^13^C HMBC spectrum of the component 3a-4; Figure S12. Comparison of ^1^H NMR spectra of the reactant 2 (red) and component 3b-1 (blue). New signals in the spectrum of 3b-1 are marked with an asterisk; Figure S13. COSY spectrum of the component 3b-1; Figure S14. ^1^H-^13^C HSQC spectrum of the component 3b-1; Figure S15. ^1^H-^13^C HMBC spectrum of the component 3b-1; Figure S16. Comparison of ^1^H NMR spectra of the macrozone 3b (blue) and component 3b-2 (red); Figure S17. ^1^H-^13^C HSQC spectrum of the component 3b-2; Figure S18. ^1^H-^13^C HMBC spectrum of the component 3b-2; Figure S19. MS spectrum of the component 3b-2; Figure S20. Comparison of ^1^H NMR spectra of the macrozone 3b (red) and component 3b-3 (blue); Figure S21. COSY spectrum of the component 3b-3; Figure S22. ^1^H-^13^C HSQC spectrum of the component 3b-3; Figure S23. ^1^H-^13^C HMBC spectrum of the component 3b-3; Figure S24. NOESY spectrum of the component 3b-3; Figure S25. MS spectrum of the component 3b-3; Figure S26. MS spectrum of the component 3a-6; Figure S27. COSY spectrum of the macrozone 3a; Figure S28. ^1^H-^13^C HSQC spectrum of the macrozone 3a; Figure S29. ^1^H-^13^C HMBC spectrum of the macrozone 3a; Figure S30. COSY spectrum of the macrozone 3a-5; Figure S31. ^1^H-^13^C HSQC spectrum of the macrozone 3a-5; Figure S32. ^1^H-^13^C HMBC spectrum of the macrozone 3a-5; Figure S33. COSY spectrum of the macrozone 3a-6; Figure S34. ^1^H-^13^C HSQC spectrum of the macrozone 3a-6; Figure S35. ^1^H-^13^C HMBC spectrum of the macrozone 3a-6; Figure S36. Distance between H-2 and H-14Me atoms during the MD simulations of compounds: 3a, 3a-5 and 3a-6. To reduce noise, each point (*d*_i_) is given as an average value of the previous and following five points ($d=\frac{\sum_{i-5}^{i+5}d_{i}}{11}$); Figure S37. Distance between H-2 and H-11 atoms during the MD simulations of compounds: 3a, 3a-5 and 3a-6. To reduce noise, each point (*d_t_*) is given as an average value of the previous and following five points (*d_t_*_−5_–*d_t_*_+5_); Figure S38. Distance between H-2Me and H-10 atoms during the MD simulations of compounds: 3a, 3a-5 and 3a-6. To reduce noise, each point (*d_t_*) is given as an average value of the previous and following five points (*d_t_*_−5_–*d_t_*_+5)_; Figure S39. Distance between H-2Me and H-3b atoms during the MD simulations of compounds: 3a, 3a-5 and 3a-6. To reduce noise, each point (*d_t_*) is given as an average value of the previous and following five points (*d_t_*_−5_–*d_t_*_+5_); Figure S40. Distance between H-2′ and H-4Me atoms during the MD simulations of compounds: 3a, 3a-5 and 3a-6. To reduce noise, each point (*d_t_*) is given as an average value of the previous and following five points (*d_t_*_−5_–*d_t_*_+5_); Figure S41. Distance between H-2′ and H-4 atoms during the MD simulations of compounds: 3a, 3a-5 and 3a-6. To reduce noise, each point (*d_t_*) is given as an average value of the previous and following five points (*d_t_*_−5_–*d_t_*_+5_); Figure S42. Distance between H-1′ and H-6Me atoms during the MD simulations of compounds 3a, 3a-5 and 3a-6. To reduce noise, each point (*d_t_*) is given as an average value of the previous and following five points (*d*_*t*−5_–*d*_*t*+5_); Figure S43. Narrow regions of the NOESY spectra of (a) 3b and (b) 3b-6 for atoms H-1′, H-2′, H-2 and H-2Me; Figure S44. Distance between atoms: (a) H-2 and H-1′; (b) H-2 and H-3b; (c) H-2 and H-4Me; (d) H-2 and H-12Me; (e) H-2Me and H-9a; (f) H-2Me and H-7; (g) H-1′ and H-3b; (h) H-1′ and H-6Me; (i) H-1′ and H-4Me; (j) H-2′ and H-4Me; (k) H-2′ and H-13 during the MD simulations of compounds 3b and 3b-6. To reduce noise, each point (*d_t_*) is given as an average value of the previous and following five points (*d*_*t*−5_–*d*_*t*+5_); Figure S45. Synthetic route for the preparation of 4′′-aminopropyl azithromycin (P5). Synthesis consists of several steps that include protection of more reactive hydroxyl groups (products P1 and P2), the addition of acrylonitrile (product P3) followed by catalytic hydrogenation (product P4) and the removal of protective groups in the final step (product P5) [7,8]; Figure S46. Synthetic route for the preparation of 3-aminopropyl azithromycin (S6). Synthesis consists of several steps that include removal of decladinose sugar unit under acidic conditions, protection of more reactive hydroxyl groups (product S1–S3), the addition of acrylonitrile (product S4) followed by catalytic hydrogenation (product S5) and the removal of protective groups in the final step (product S6) [7,8]; Figure S47. Synthetic route for the preparation of thiosemicarbazones. The first step involves a reaction between isothiocyanate and hydrazine monohydrate to form thiosemicarbazide (product A). The second step includes the reaction of thiosemicarbazide and 4-formylbenzoic acid to form thiosemicarbazone (product B) [7,8]; Table S1. Vicinal coupling constants ^3^*J*_H2_,_H3_ and NOE proton–proton contacts of isomers of the macrozone 3a and 3b; Table S2. Monitored distances around C-2′ atom during the 500 ns of molecular dynamics simulations for different diastereomeric configurations; Table S3. Monitored distances around C-2′ atom during the 500 ns of molecular dynamics simulations for different diastereomeric configurations.
