# Supplementary material for: Screening of Novel Antimicrobial Diastereomers of Azithromycin–Thiosemicarbazone Conjugates: A Combined LC-SPE/Cryo NMR, MS/MS and Molecular Modeling Approach
Source: Antibiotics (Basel). 2022 Dec 2;11(12):1738. doi: 10.3390/antibiotics11121738 (PMC9774193; doi:10.3390/antibiotics11121738)
Supplement: Supplementary file 1 [file antibiotics-11-01738-s001.zip › antibiotics-2059833-supplementary.pdf]

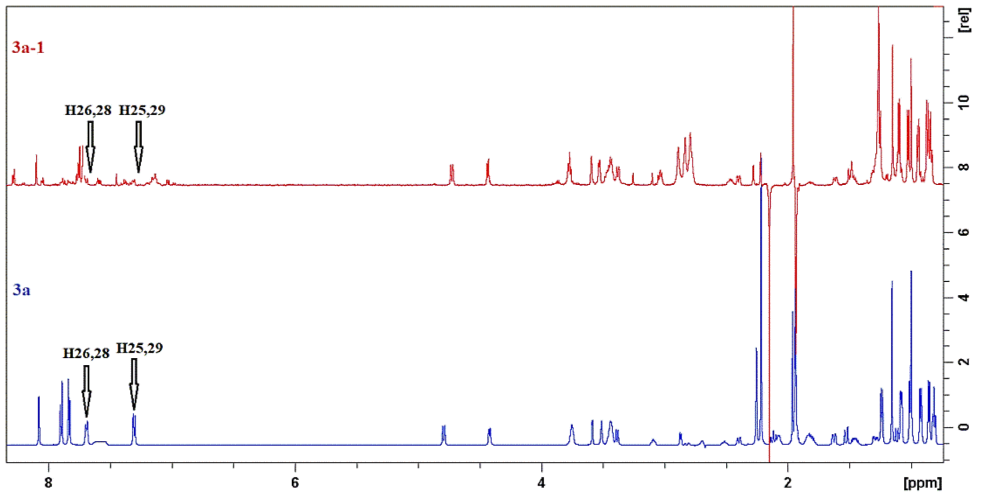

Figure S1. Comparison of ^{1}H NMR spectra of the macrozone **3a** (blue) and component **3a-1** (red).

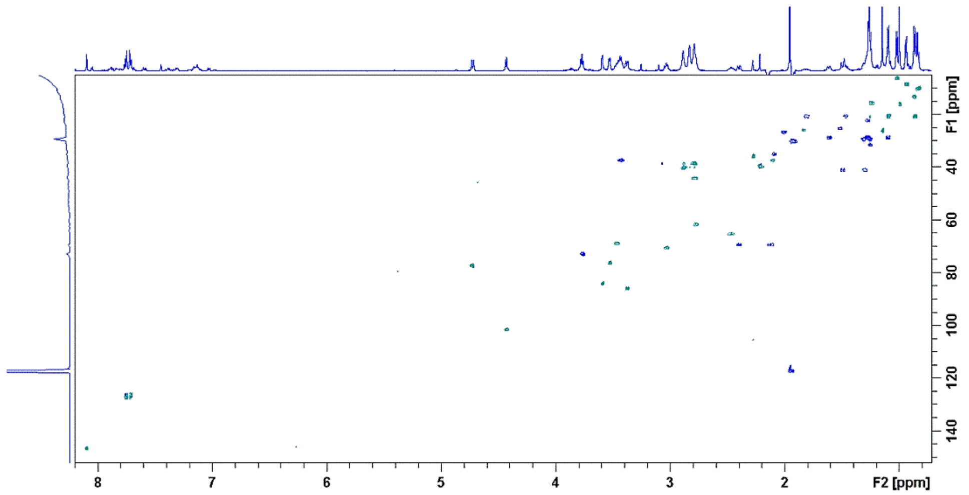

Figure S2. ^{1}H-^{13}C HSQC spectrum of the component **3a-1**.

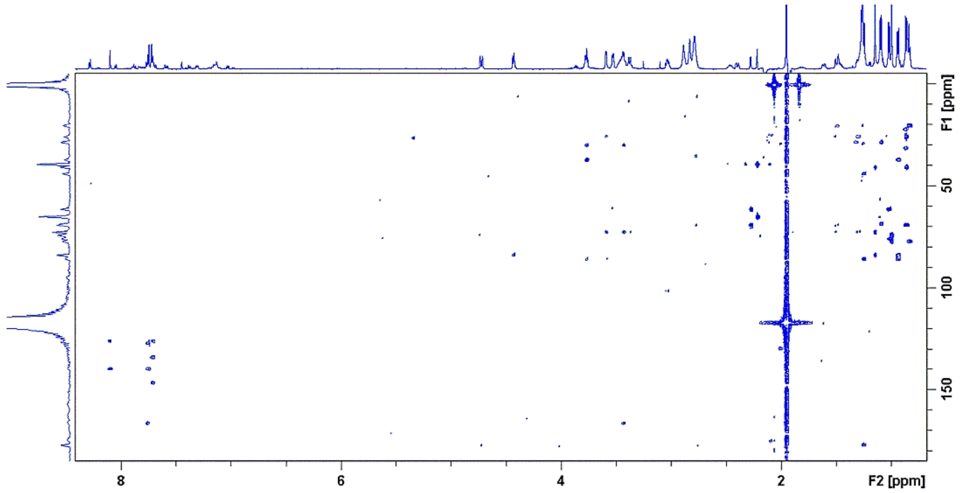

Figure S3. ^{1}H-^{13}C HMBC spectrum of the component **3a-1**.

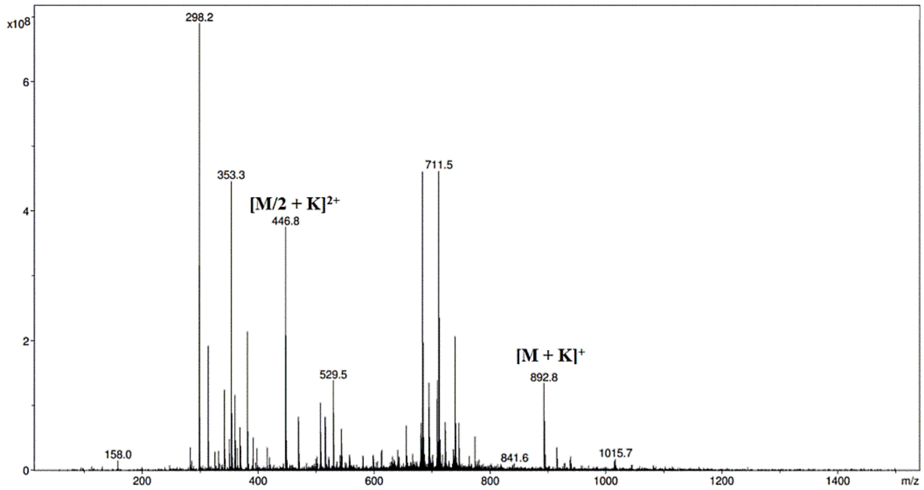

Figure S4. MS spectrum of the component **3a-1**.

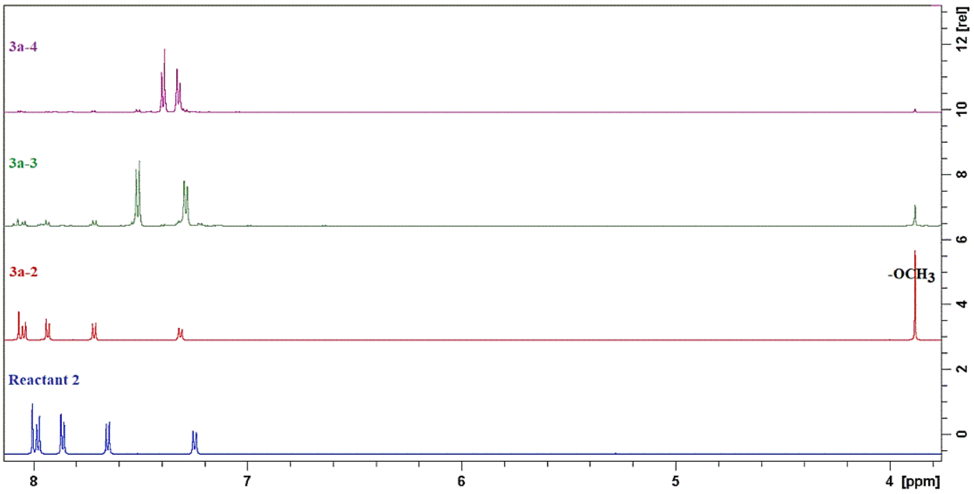

Figure S5. Comparison of ^{1}H NMR spectra of the reactant **2** (blue), component **3a-2** (red), component **3a-3** (green) and component **3a-4** (purple).

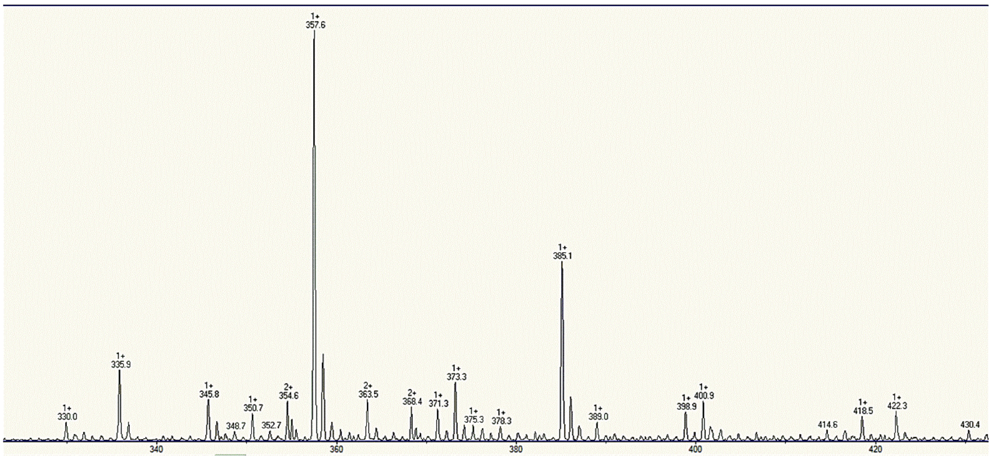

Figure S6. MS spectrum of the component **3a-2**.

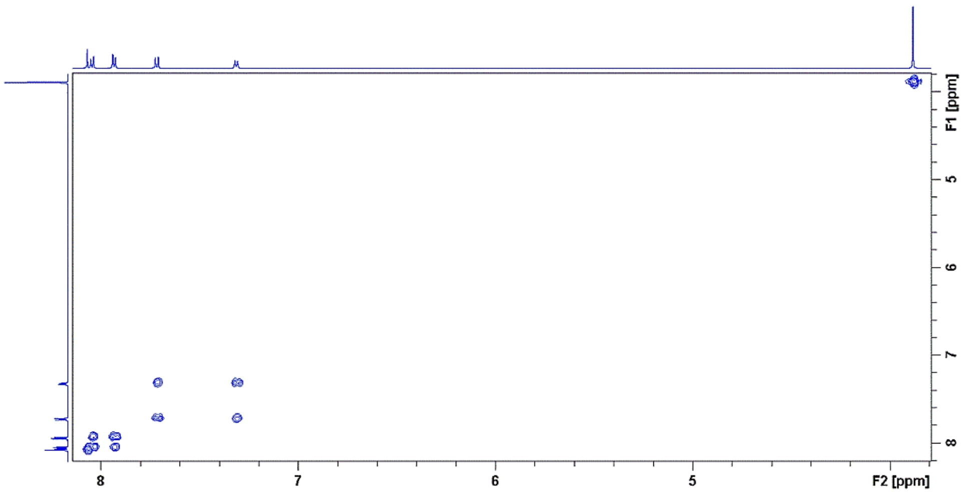

Figure S7. COSY spectrum of the component **3a-2**.

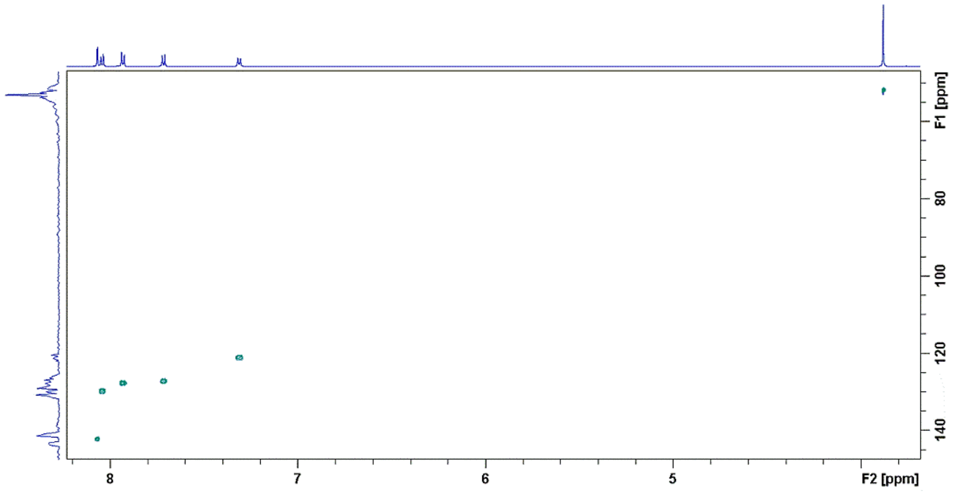

Figure S8. ^{1}H-^{13}C HSQC spectrum of the component **3a-2**.

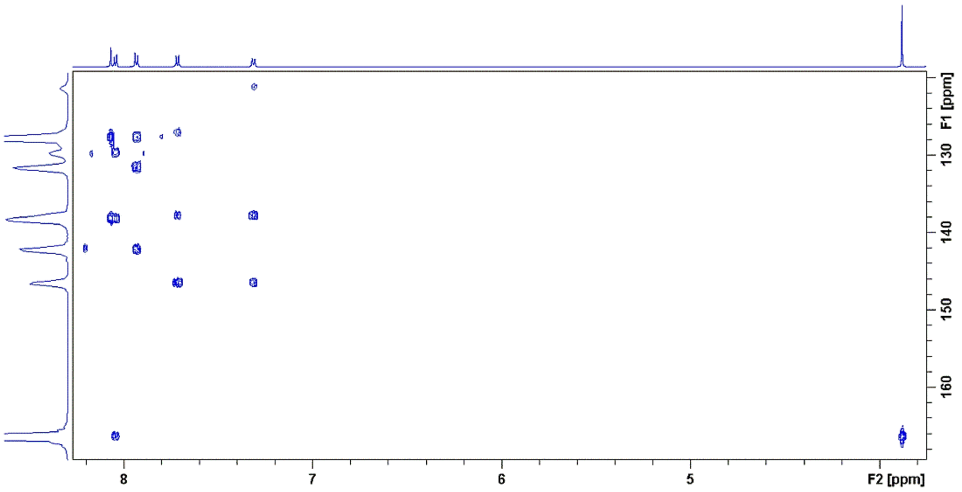

Figure S9. ^{1}H-^{13}C HMBC spectrum of the component **3a-2**.

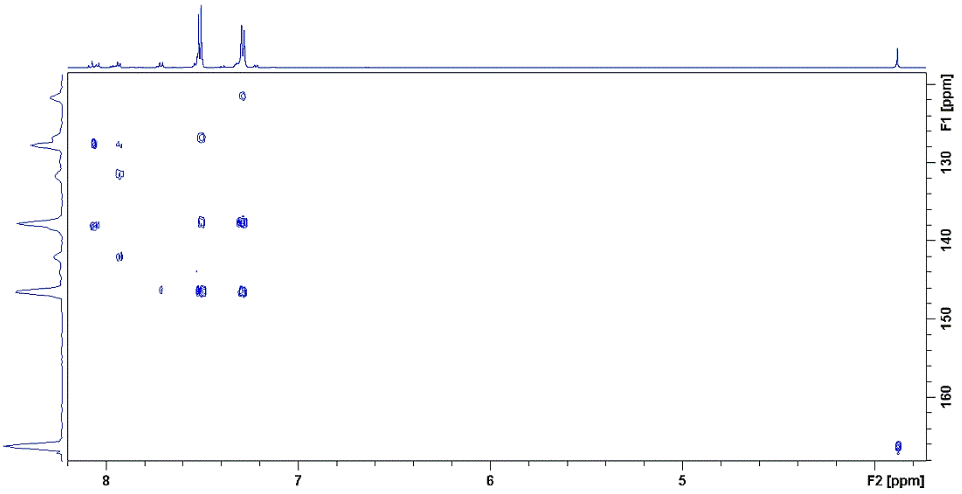

Figure S10. ^{1}H-^{13}C HMBC spectrum of the component **3a-3**.

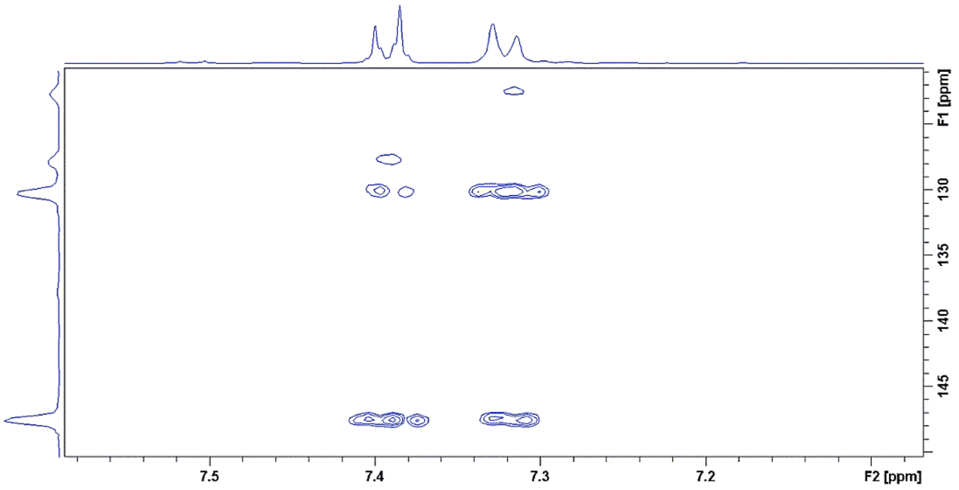

Figure S11. ^{1}H-^{13}C HMBC spectrum of the component **3a-4**.

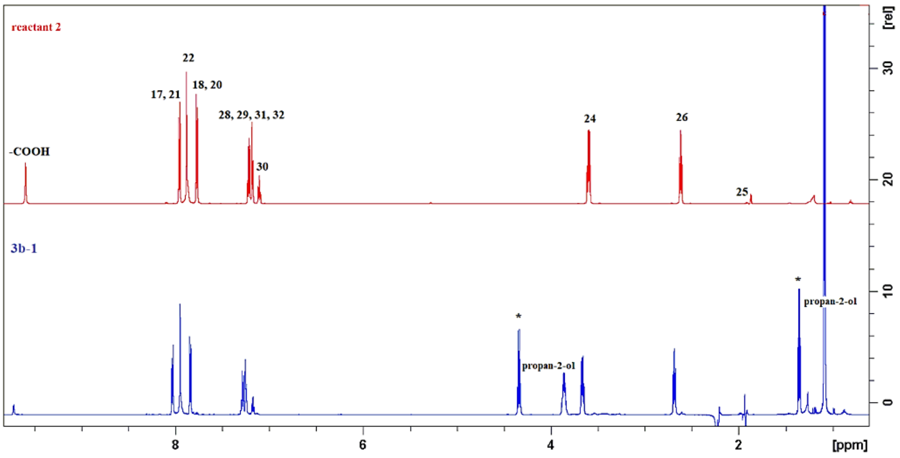

Figure S12. Comparison of ^{1}H NMR spectra of the reactant **2** (red) and component **3b-1** (blue). New signals in the spectrum of **3b-1** are marked with asterisk.

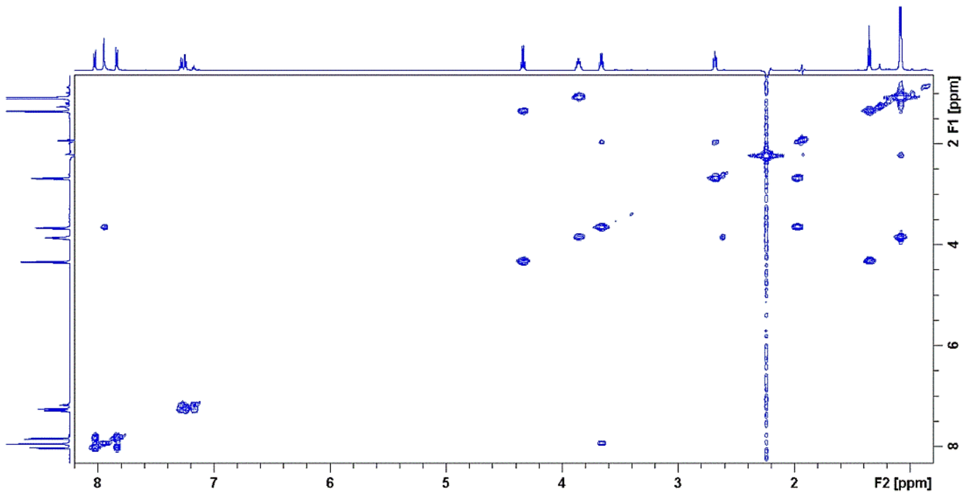

Figure S13. COSY spectrum of the component **3b-1**.

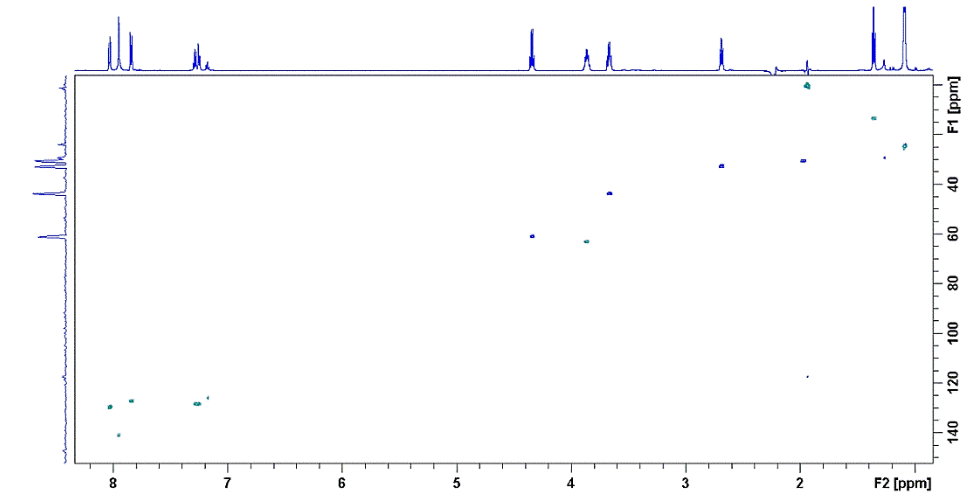

Figure S14. ^{1}H-^{13}C HSQC spectrum of the component **3b-1**.

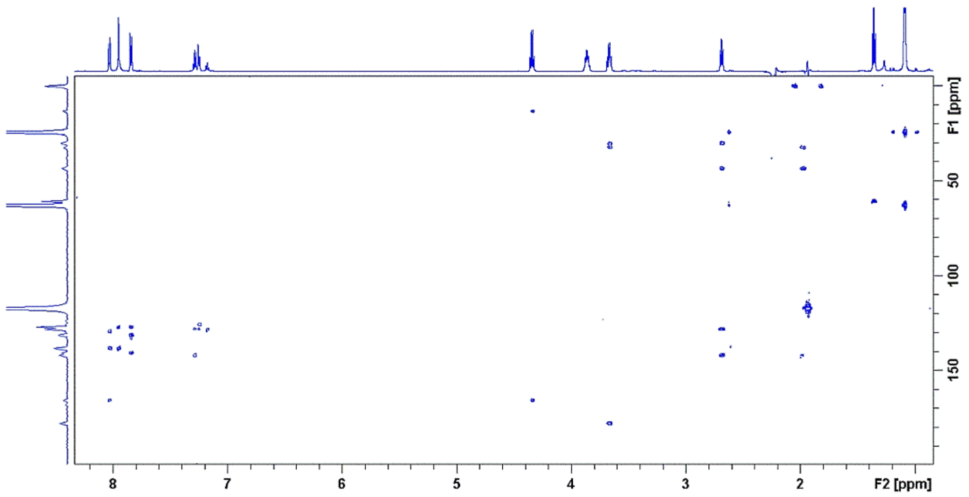

Figure S15. ^{1}H-^{13}C HMBC spectrum of the component **3b-1**.

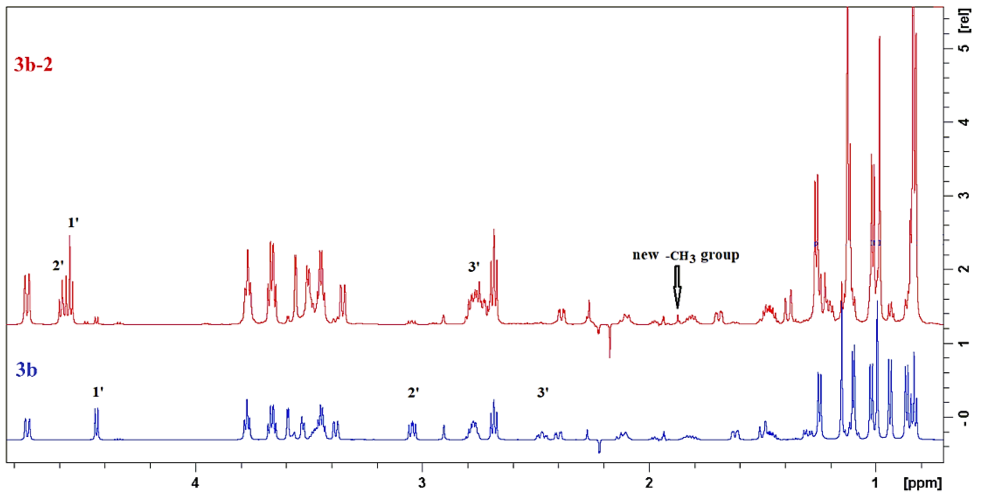

Figure S16. Comparison of ^1H NMR spectra of the macrozone **3b** (blue) and component **3b-2** (red).

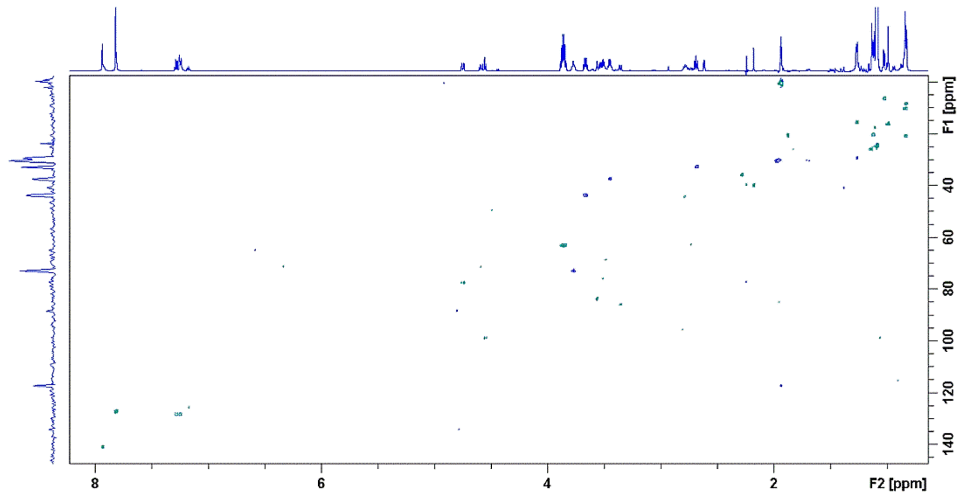

Figure S17. ^1H-^{13}C HSQC spectrum of the component **3b-2**.

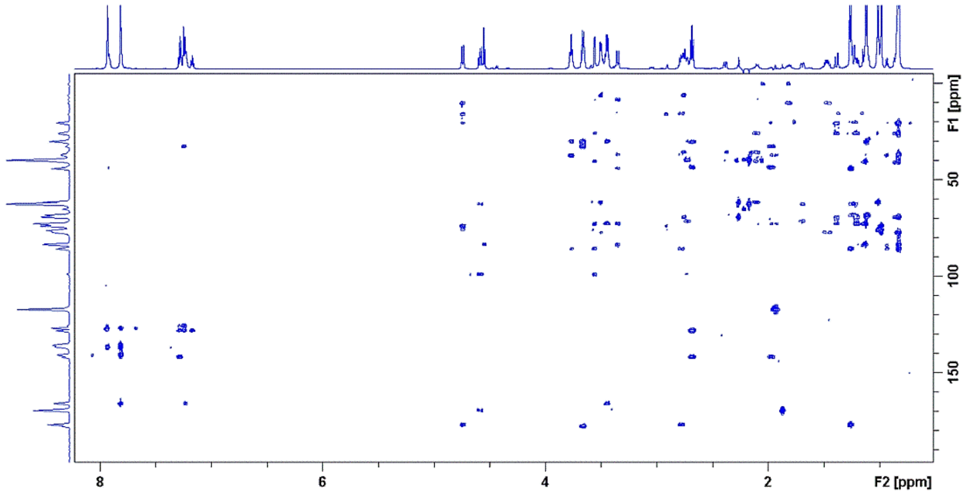

Figure S18. ^1H-^{13}C HMBC spectrum of the component **3b-2**.

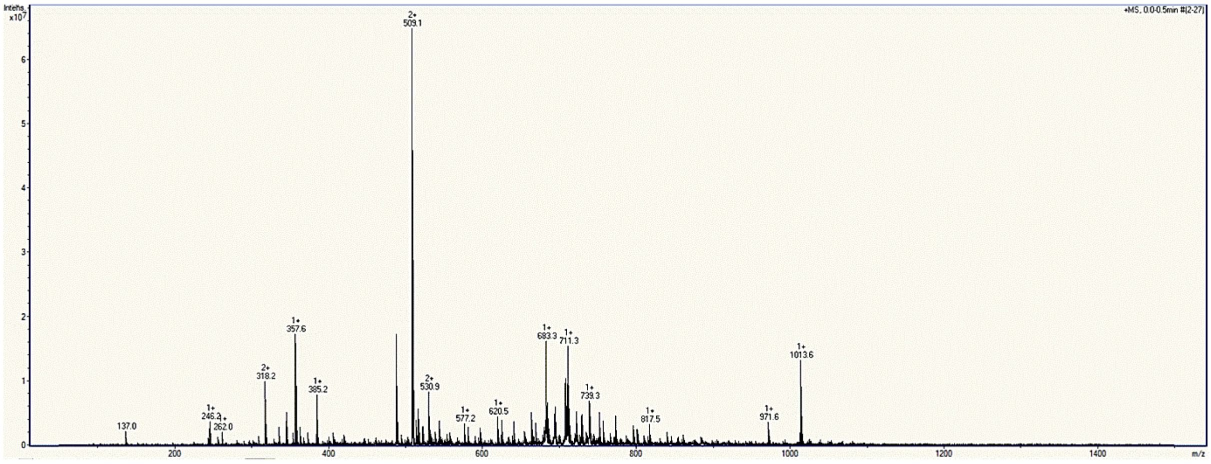

Figure S19. MS spectrum of the component **3b-2**.

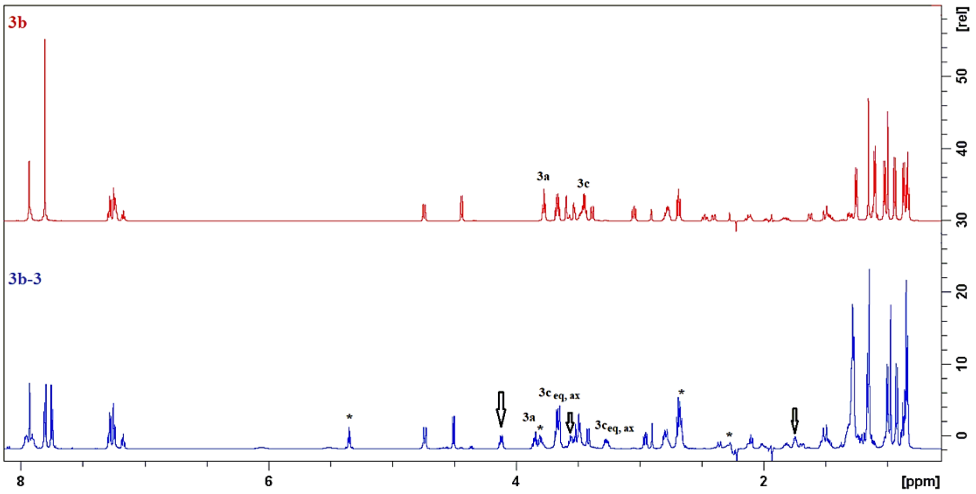

Figure S20. Comparison of ^{1}H NMR spectra of the macrozone **3b** (red) and component **3b-3** (blue).Signals of the ethylene moiety -CH_2-CH_2-R in the spectrum of **3b-3** are marked with an arrow. Signals of the unknown impurity are marked with asterisk.

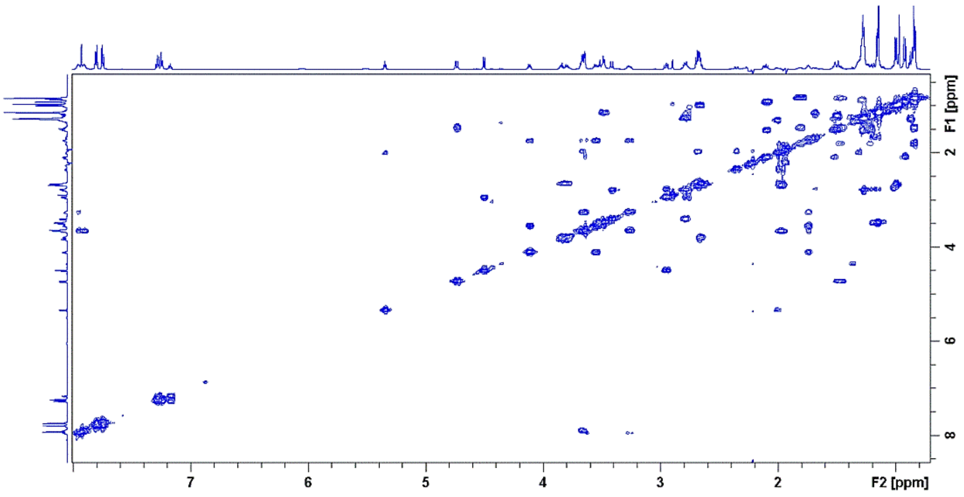

Figure S21. COSY spectrum of the component **3b-3**.

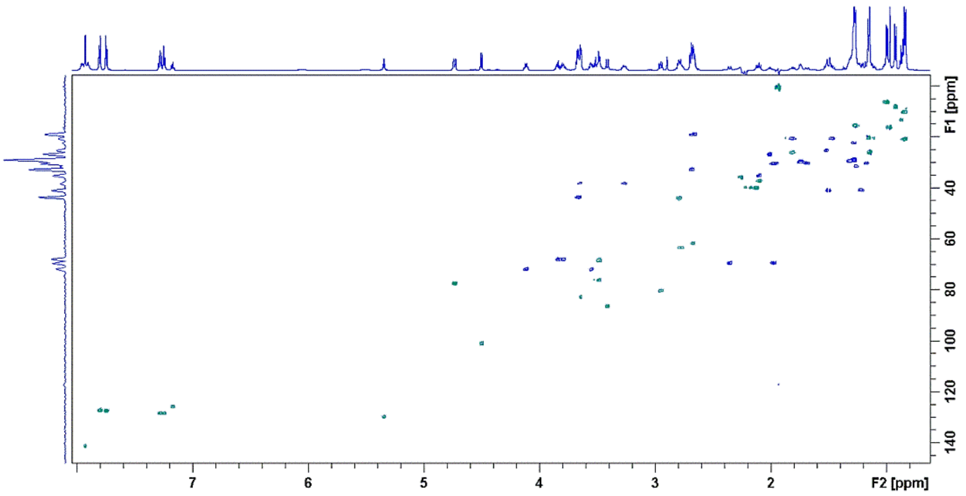

Figure S22. ^{1}H-^{13}C HSQC spectrum of the component **3b-3**.

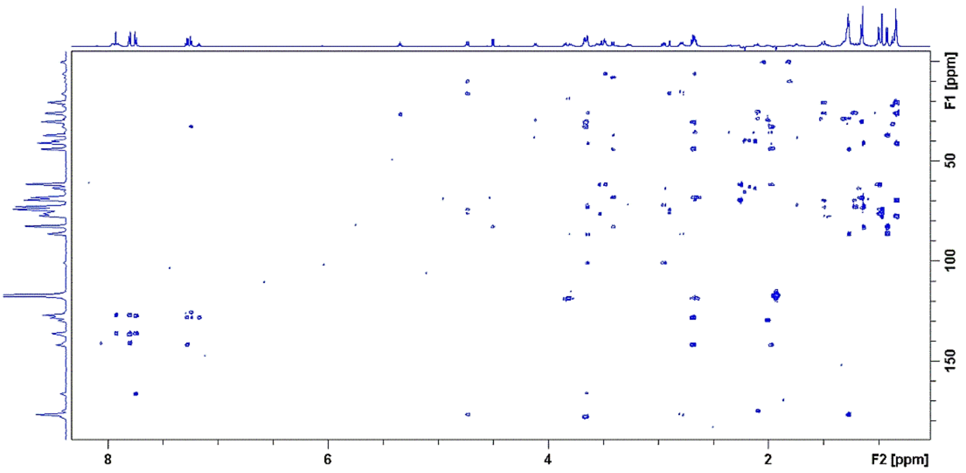

Figure S23. ^{1}H-^{13}C HMBC spectrum of the component **3b-3**.

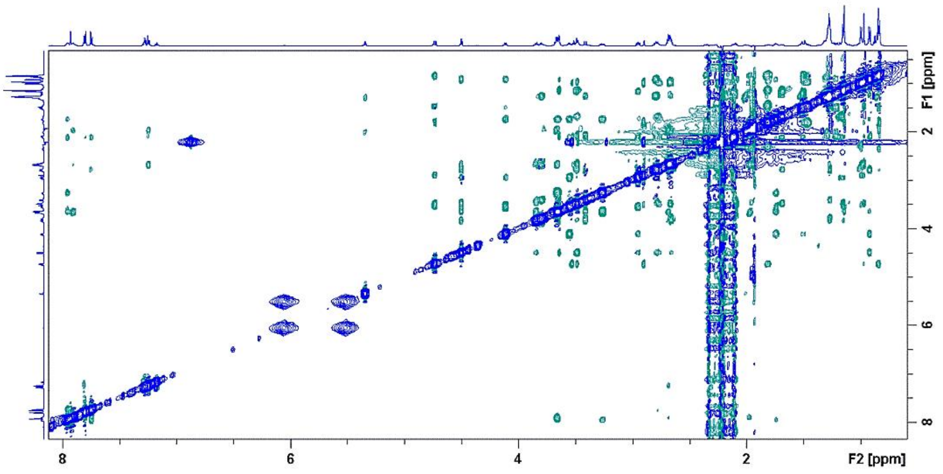

Figure S24. NOESY spectrum of the component **3b-3**.

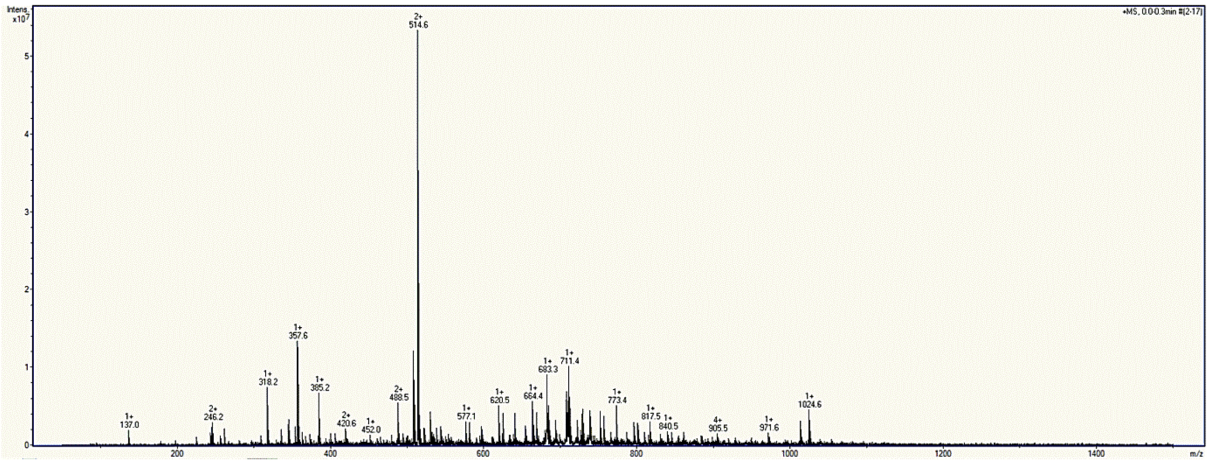

Figure S25. MS spectrum of the component **3b-3**.

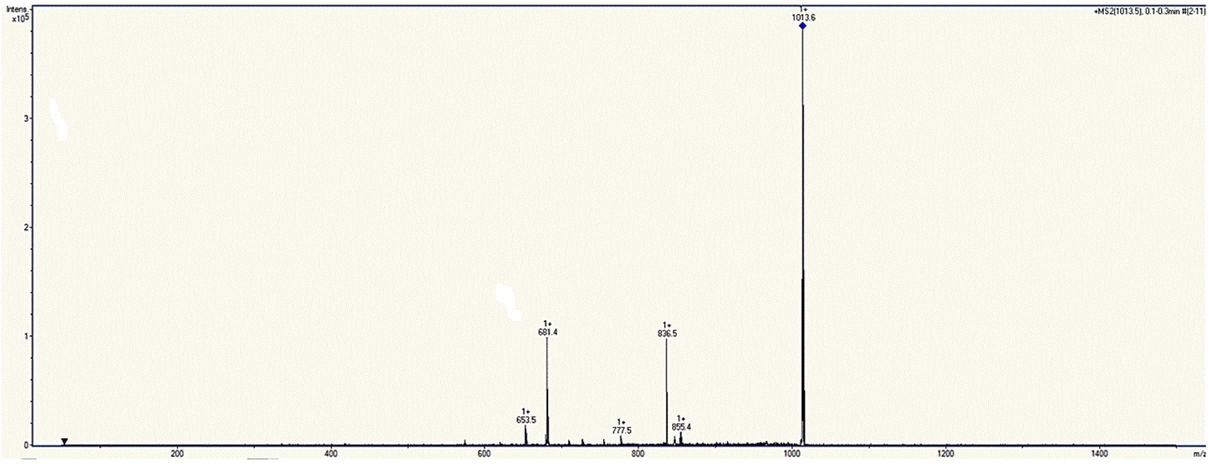

Figure S26. MS spectrum of the component **3a-6**.

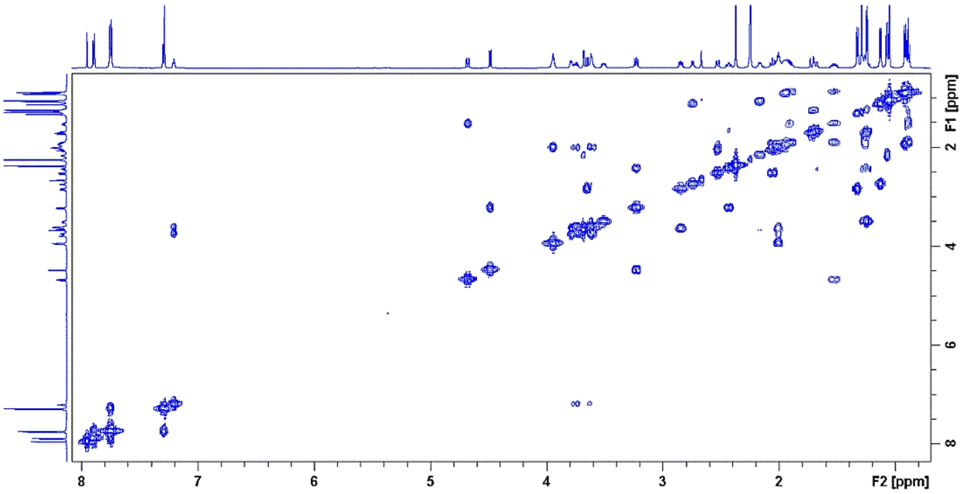

Figure S27. COSY spectrum of the macrozone **3a**.

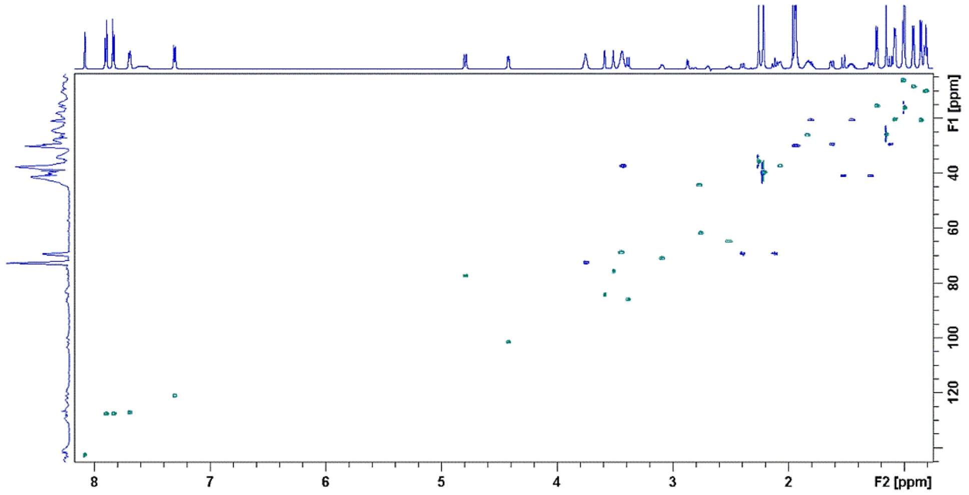

Figure S28. ^{1}H-^{13}C HSQC spectrum of the macrozone **3a**.

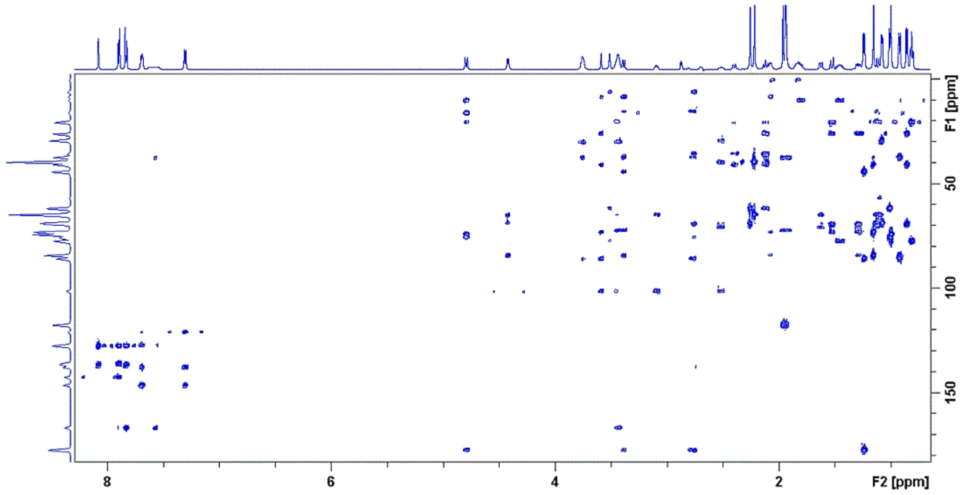

Figure S29. ^{1}H-^{13}C HMBC spectrum of the macrozone **3a**.

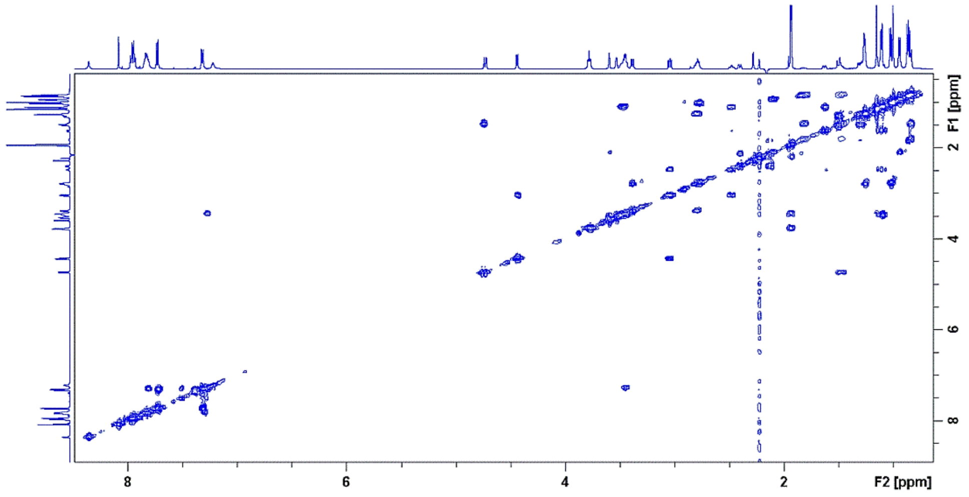

Figure S30. COSY spectrum of the macrozone **3a-5**.

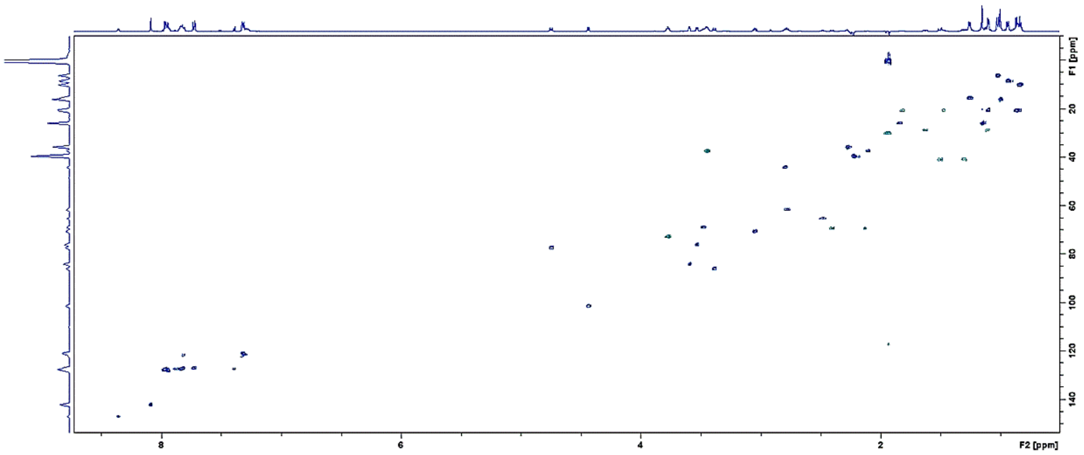

Figure S31. ^{1}H-^{13}C HSQC spectrum of the macrozone **3a-5**.

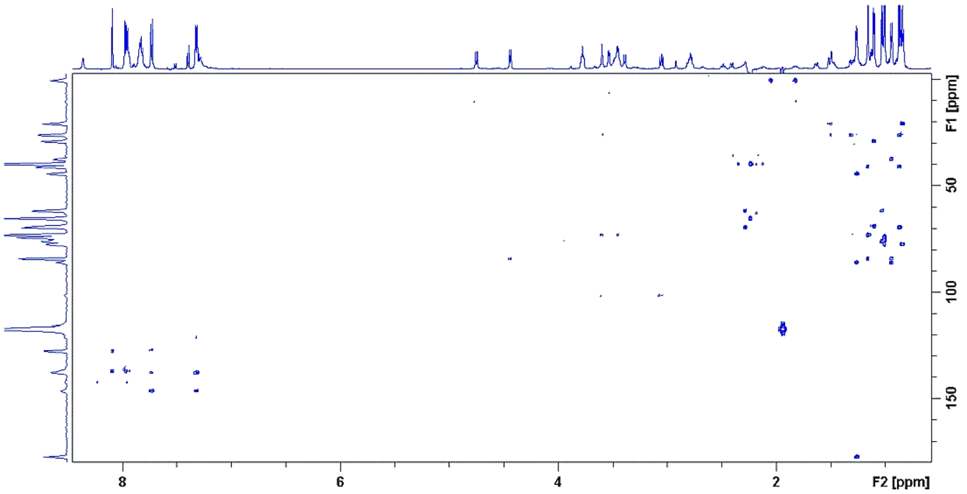

Figure S32. ^{1}H-^{13}C HMBC spectrum of the macrozone **3a-5**.

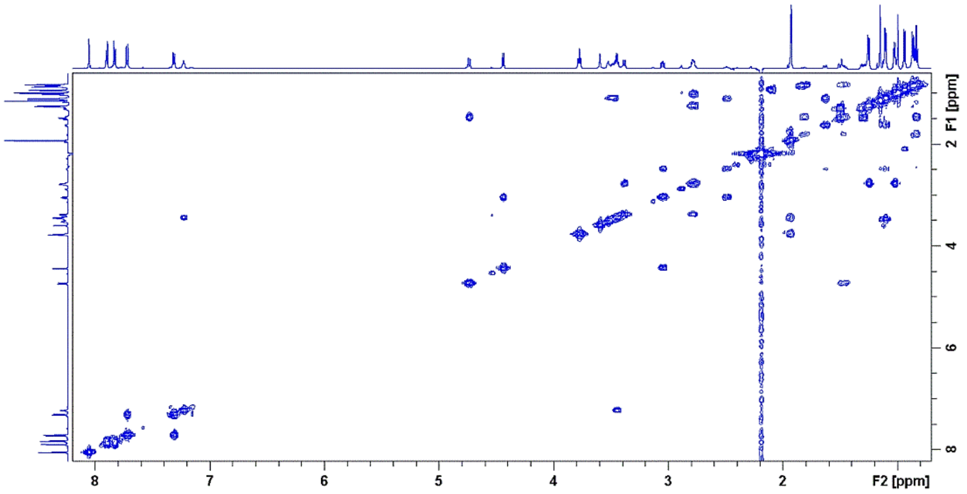

Figure S33. COSY spectrum of the macrozone **3a-6**.

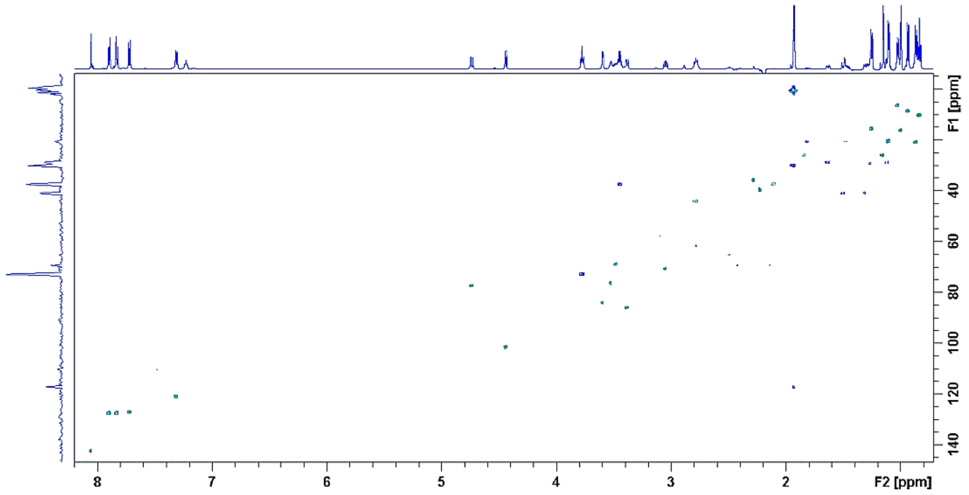

Figure S34. ^{1}H-^{13}C HSQC spectrum of the macrozone **3a-6**.

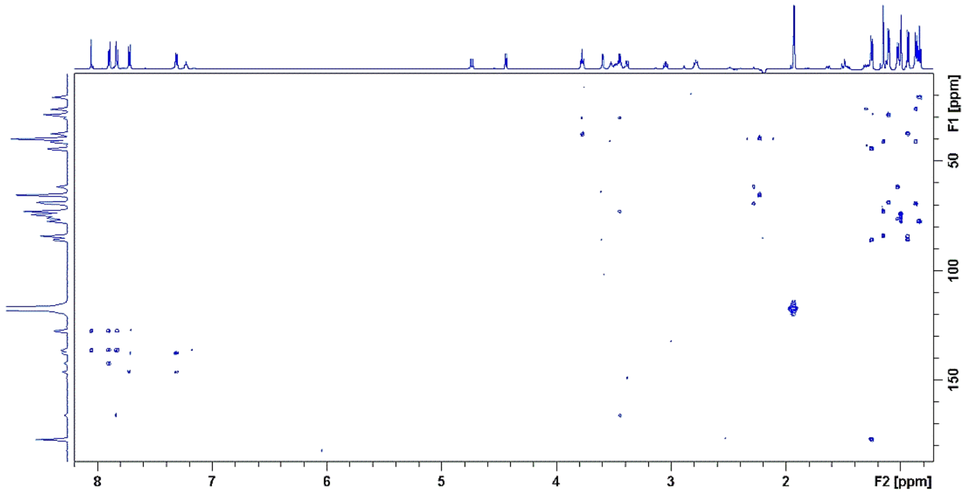

Figure S35. ^{1}H-^{13}C HMBC spectrum of the macrozone **3a-6**.

Table S1. Vicinal coupling constants $^{3}J_{H2,H3}$ and NOE proton-proton contacts of isomers of the macrozone **3a** and **3b**.

Compound	$^{3}J_{H2,H3}$ / Hz	H4-H11	H3-H11
3a	10,12	+	-
3b	10,05	+	-
3a-5	10,31	noise	-
3b-6	10,01	+	-
3a-6	10,07	+	-

Table S2. Monitored distances around C-2 atom during the 500 ns of molecular dynamics simulations for different diastereomeric configurations.

Compound	Configuration of C-2 atom	Distance / nm					
		H2-H14Me	H2-H11	H2Me-H7	H2Me-H9a	H2Me-H10	H2Me-H3b
**3a**	R	4.2 ± 0.4	5.6 ± 0.3	6.6 ± 0.3	8.6 ± 0.3	8.2 ± 0.2	4.4 ± 0.3
**3a-5**	S	5.2 ± 0.3	7.1 ± 0.2	4.7 ± 0.3	4.0 ± 0.4	4.4 ± 0.7	5.9 ± 0.3
**3a-6**	R	4.3 ± 0.3	4.5 ± 0.3	6.8 ± 0.2	9.0 ± 0.3	7.9 ± 0.2	4.4 ± 0.3

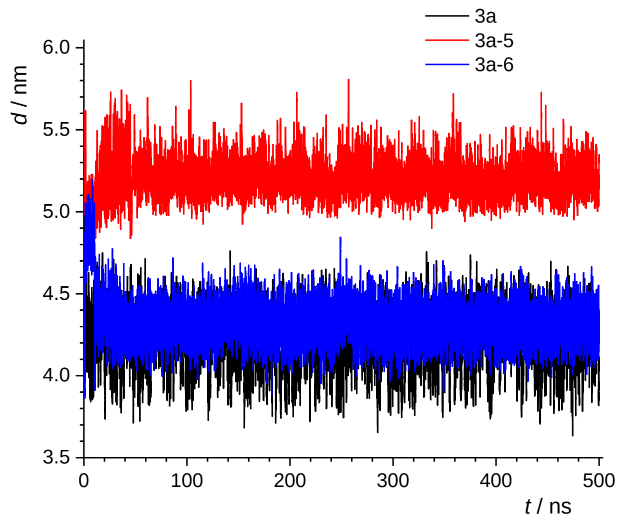

Figure S36. Distance between H-2 and H-14Me atoms during the MD simulations of compounds: **3a**, **3a-5** and **3a-6**. To reduce noise, each point (d_i) is given as an average value of previous and following five points ($d = \frac{\sum_{i-5}^{i+5} d_i}{11}$).

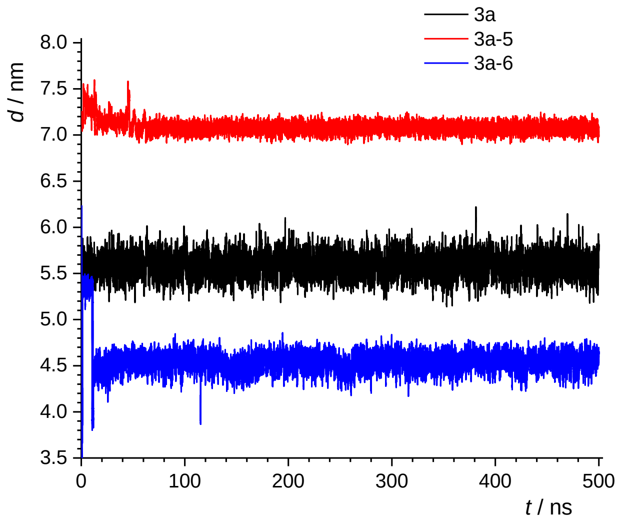

Figure S37. Distance between H-2 and H-11 atoms during the MD simulations of compounds: **3a**, **3a-5** and **3a-6**. To reduce noise, each point (d_t) is given as an average value of previous and following five points ($d_{t-5} - d_{t+5}$).

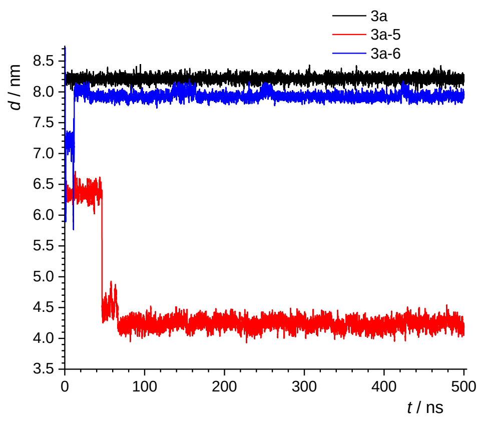

Figure S38. Distance between H-2Me and H-10 atoms during the MD simulations of compounds: **3a**, **3a-5** and **3a-6**. To reduce noise, each point (d_t) is given as an average value of previous and following five points ($d_{t-5} – d_{t+5}$).

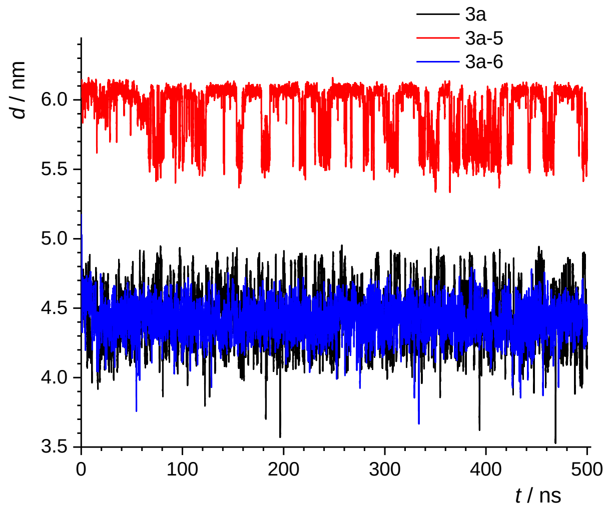

Figure S39. Distance between H-2Me and H-3b atoms during the MD simulations of compounds: **3a**, **3a-5** and **3a-6**. To reduce noise, each point (d_t) is given as an average value of previous and following five points ($d_{t-5} – d_{t+5}$).

Table S3. Monitored distances around C-2′ atom during the 500 ns of molecular dynamics simulations for different diastereomeric configurations.

Compound	Configuration of C-2′ atom	Distance / nm	
		H2'-H4Me	H2'-H4
**3a**	R	5.0 ± 0.2	5.2 ± 0.2
**3a-5**	S	4.4 ± 0.4	4.5 ± 0.4
**3a-6**	R	4.7 ± 0.2	5.4 ± 0.2

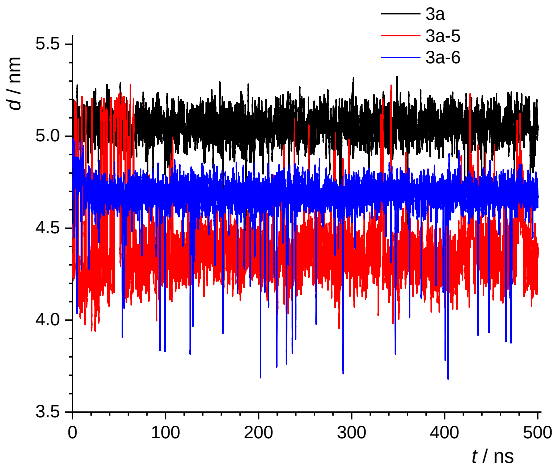

Figure S40. Distance between H-2′ and H-4Me atoms during the MD simulations of compounds: **3a**, **3a-5** and **3a-6**. To reduce noise, each point (d_t) is given as an average value of previous and following five points ($d_{t-5} – d_{t+5}$).

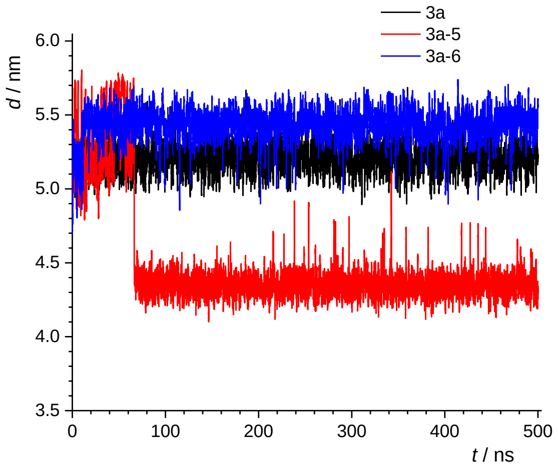

Figure S41. Distance between H-2′ and H-4 atoms during the MD simulations of compounds: **3a**, **3a-5** and **3a-6**. To reduce noise, each point (d_t) is given as an average value of previous and following five points ($d_{t-5} – d_{t+5}$).

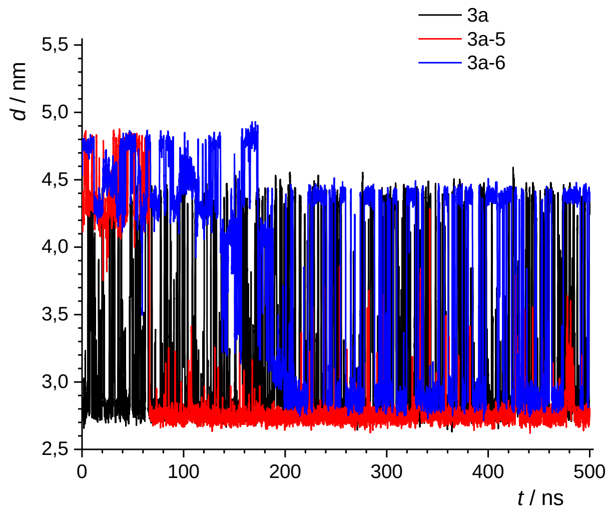

Figure S42. Distance between H-1' and H-6Me atoms during the MD simulations of compounds **3a**, **3a-5** and **3a-6**. To reduce noise, each point (d_t) is given as an average value of previous and following five points ($d_{t-5} - d_{t+5}$).

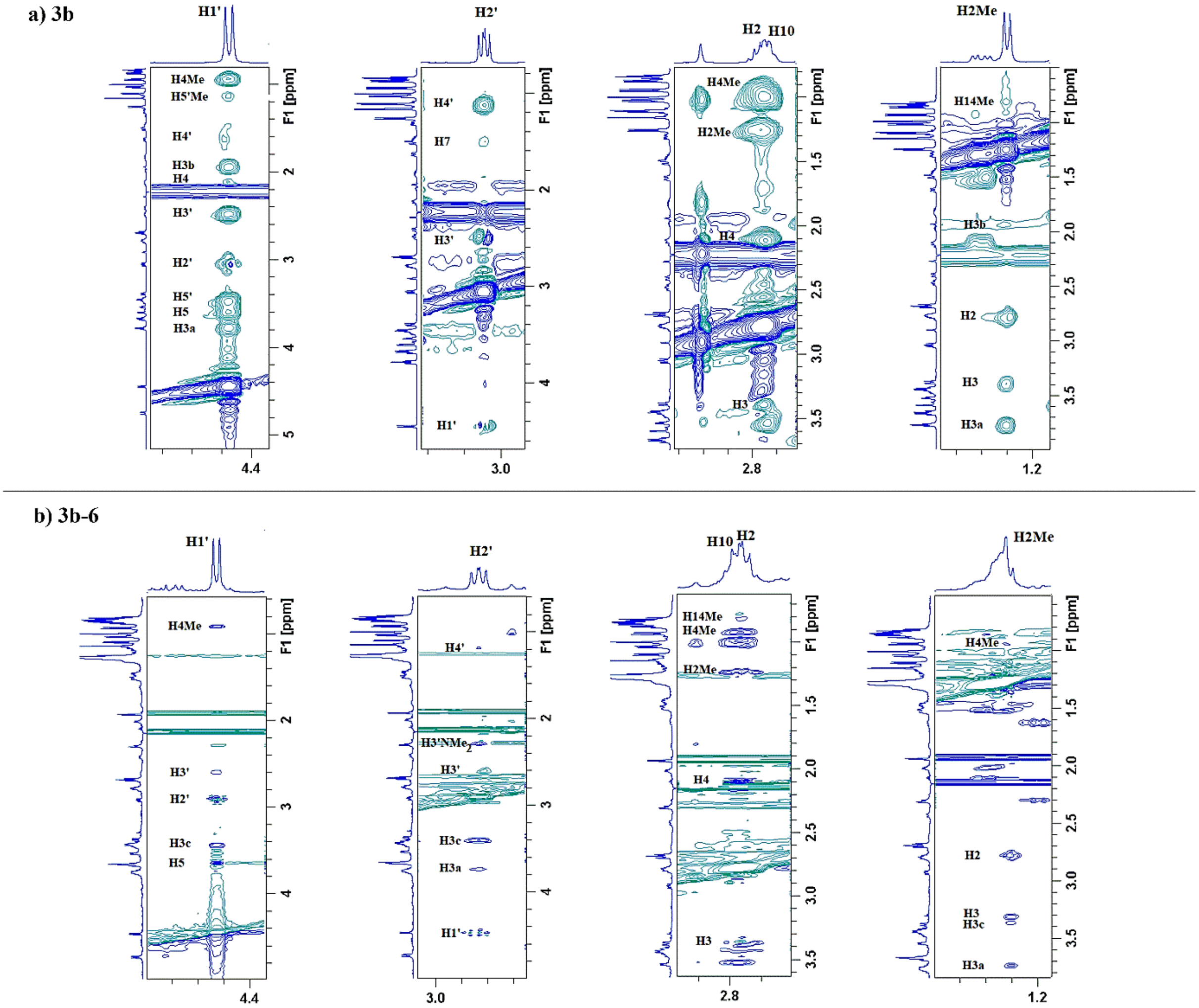

Figure S43. Narrow regions of the NOESY spectra of a) **3b** and b) **3b-6** for atoms H-1′, H-2′, H-2 and H-2Me.

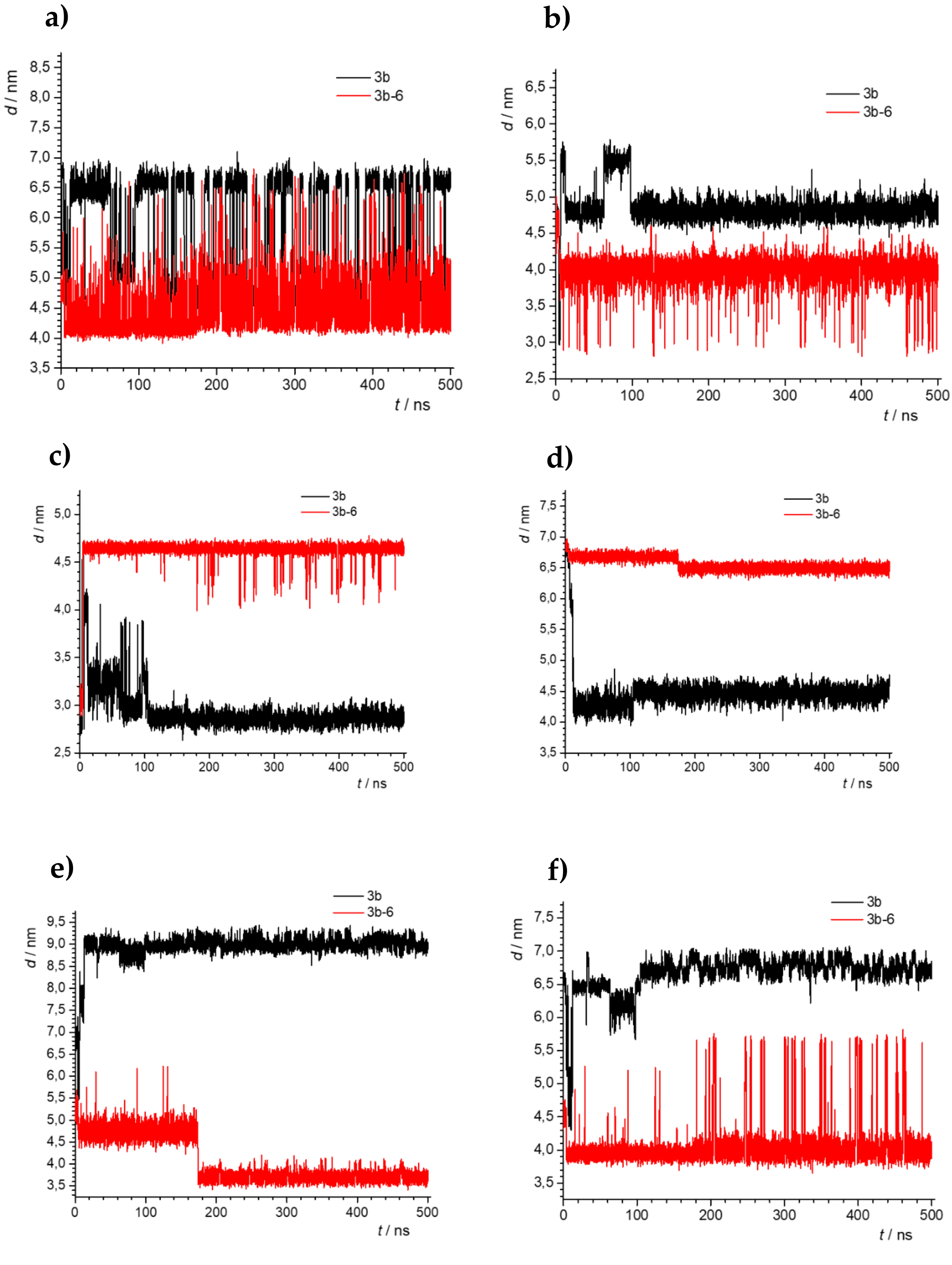
a)
b)
c)
d)
e)
f)
3b
3b-6
d / nm
t / ns

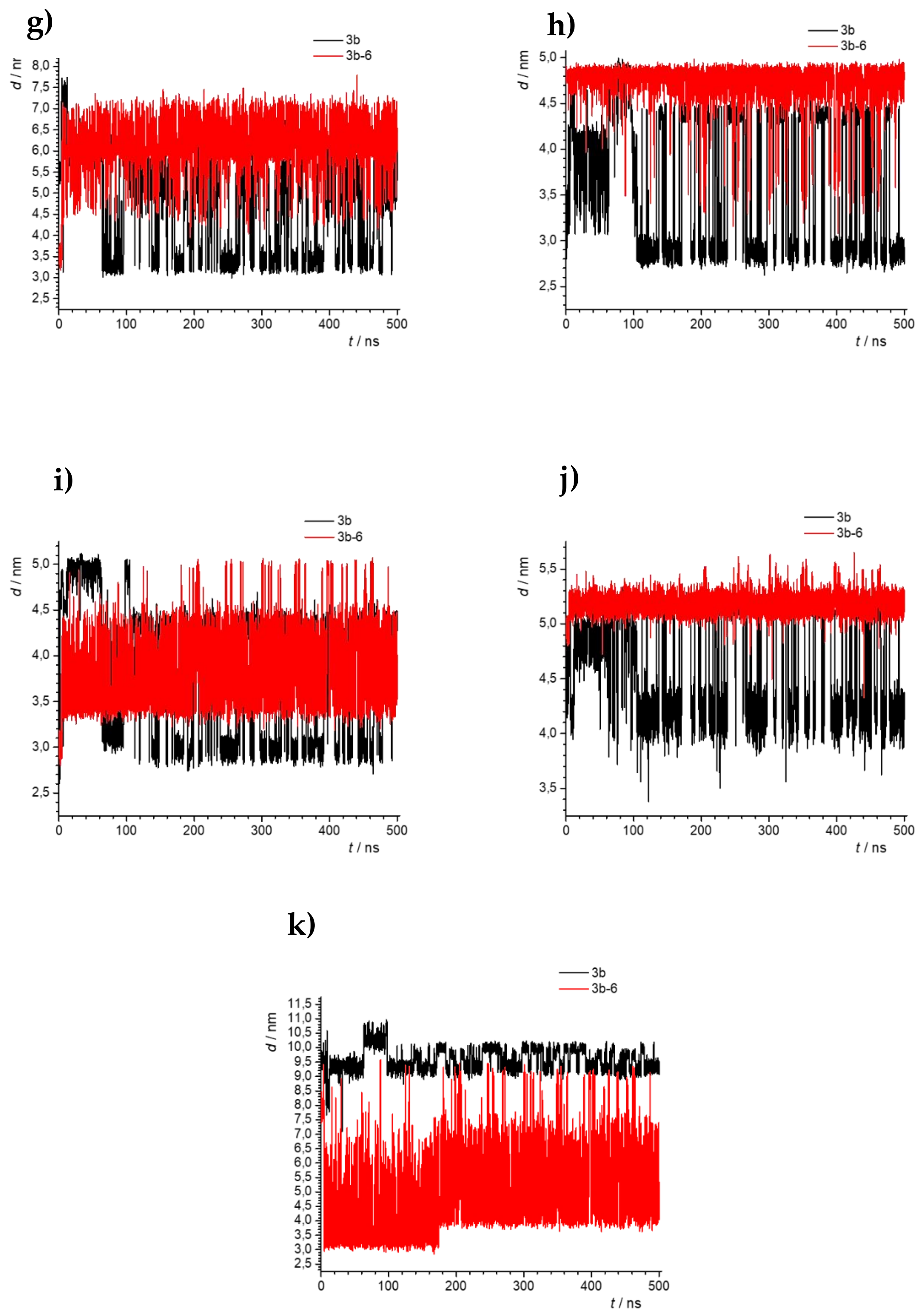

Figure S44. Distance between atoms: **a)** H-2 and H-1'; **b)** H-2 and H-3b; **c)** H-2 and H-4Me; **d)** H-2 and H-12Me; **e)** H-2Me and H-9a; **f)** H-2Me and H-7; **g)** H-1' and H-3b; **h)** H-1' and H-6Me; **i)** H-1' and H-4Me; **j)** H-2' and H-4Me; **k)** H-2' and H-13 during the MD simulations of compounds 3b and 3b-6. To reduce noise, each point (d_t) is given as an average value of previous and following five points (d_{t-5} – d_{t+5}).

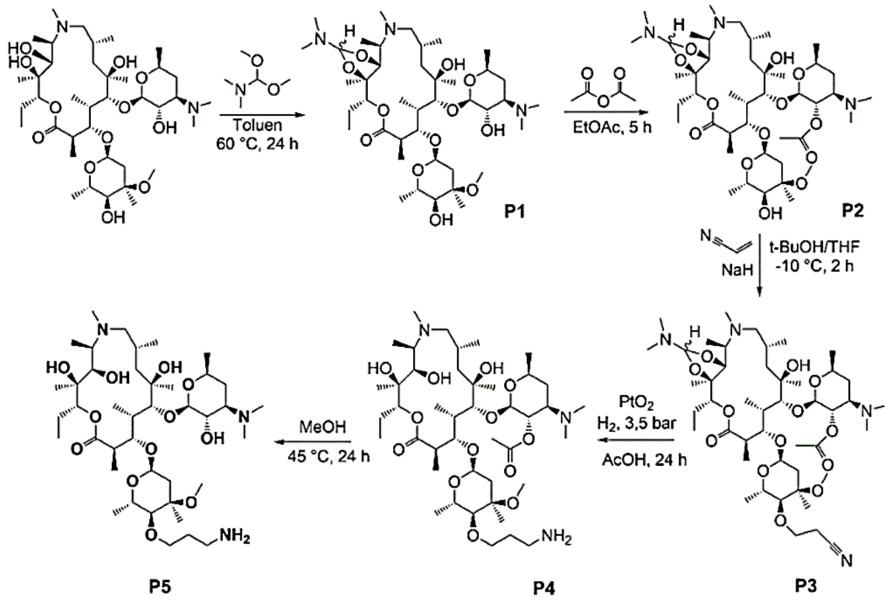

Figure S45. Synthetic route for the preparation of 4''-aminopropyl azithromycin (**P5**). Synthesis constits of several steps that include protection of more reactive hydroxyl groups (products **P1** and **P2**), the addition of acrylonitrile (product **P3**) followed by catalytic hydrogenation (product **P4**) and the removal of protective groups in the final step (product **P5**) [7-8].

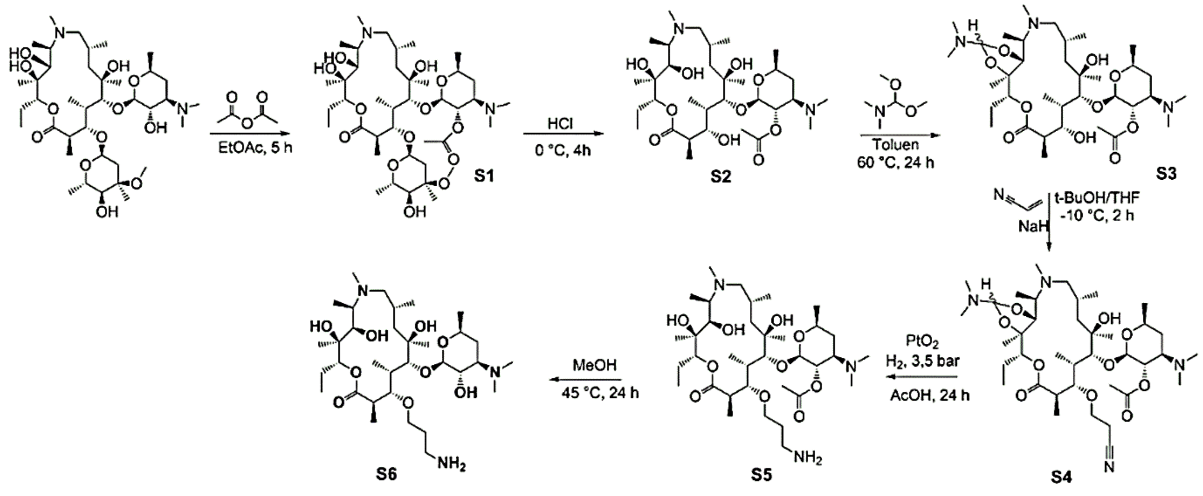

Figure S46. Synthetic route for the preparation of 3-aminopropyl azithromycin (**S6**). Synthesis constits of several steps that include removal of decladinose sugar unit under acidic conditions, protection of more reactive hydroxyl groups (product **S1-S3**), the addition of acrylonitrile (product **S4**) followed by catalytic hydrogenation (product **S5**) and the removal of protective groups in the final step (product **S6**) [7-8].

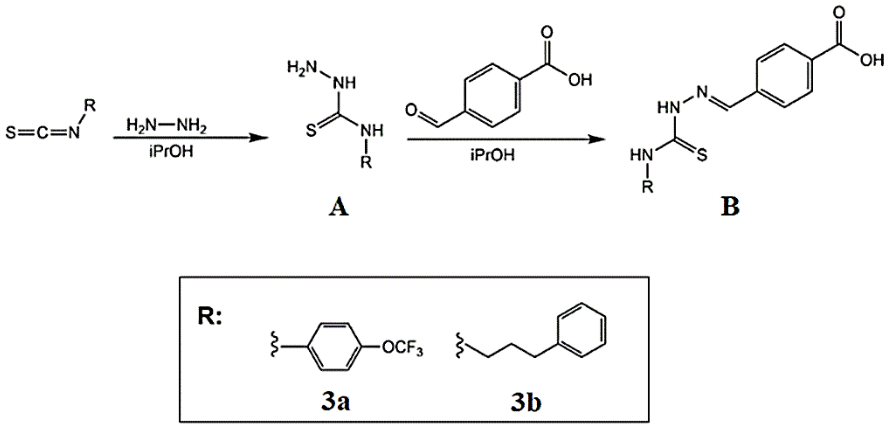

Figure S47. Synthetic route for the preparation of thiosemicarbazones. The first step involves a reaction between isothiocyanate and hydrazine monohydrate to form thiosemicarbazide (product **A**). The second step includes the reaction of thiosemicarbazide and 4-formylbenzoic acid to form thiosemicarbazone (product **B**) [7-8].
